# How developmental stressors affect mitochondrial respiratory function: a systematic review and meta‐analysis

**DOI:** 10.1002/brv.70199

**Published:** 2026-07-14

**Authors:** Ondi L. Crino, Christopher R. Friesen, Geoffrey E. Hill, Dalton C. Leibold, Pablo Recio, Karine Salin, Kristoffer H. Wild, Daniel W.A. Noble

**Affiliations:** ^1^ College of Science and Engineering Flinders University Bedford Park SA 5001 Australia; ^2^ Division of Ecology and Evolution, Research School of Biology The Australian National University Canberra ACT Australia; ^3^ School of Science, Faculty of Science, Medicine and Health University of Wollongong NSW Australia; ^4^ Department of Biological Sciences Auburn University Auburn AL USA; ^5^ Université de Brest, Ifremer, CNRS, IRD, Laboratory of Environmental Marine Sciences Plouzané France; ^6^ School of BioSciences University of Melbourne Parkville VIC Australia

**Keywords:** cellular respiration, corticosterone, cortisol, developmental programming, glucocorticoids, mitochondria, nutrition, oxidative phosphorylation, stress

## Abstract

The environmental conditions organisms experience during early development can have powerful and sustained effects on morphology, physiology, behaviour, and performance. Such developmental effects can influence reproductive success, survival, and life‐history strategies and can be transmitted across generations (i.e. trans and intergenerational effects). In this way, developmental effects can be powerful drivers of evolutionary change. Given the developmental environment affects a range of phenotypic traits, it has been proposed that physiological responses to developmental conditions are modulated through cellular mechanisms that are shared across cell and tissue types, such as mitochondrial function. Mitochondrial respiratory function is highly sensitive to environmental conditions and exposure to adverse conditions during development can have sustained effects on different aspects of aerobic respiration in mitochondria. However, it is currently unknown if these effects are widespread across taxonomic groups and which components of mitochondrial respiratory function are most likely to be affected by the environment during development. We compiled data from 86 studies to examine the effects of developmental stressors (nutritional imbalance, glucocorticoid hormone exposure, parental care deprivation, and psychological disturbance) on mitochondrial respiratory function using meta‐analysis. We sought to uncover whether there are general effects of developmental stressors on different aspects of mitochondrial respiratory function (antioxidants, metabolic capacity, oxidative damage, oxidative stress, and aerobic respiration). We tested how the type of developmental stressor, together with timing of exposure (prenatal *versus* postnatal), and sex and taxon of the test subjects influenced the magnitude, direction, and duration of effects on mitochondrial respiratory function. Finally, we tested which aspects of mitochondrial respiratory function were most impacted by developmental stressors. We found that exposure to glucocorticoids, parental care deprivation, and psychological disturbances during development generally decreased mitochondrial respiratory function. Generally, these developmental stressors increased the production of reactive oxygen species and oxidative damage and reduced aerobic respiration, metabolic capacity, and antioxidant levels. Nutritional imbalances during development (including both restricted and excessive nutrition) had a slight negative effect on mitochondrial respiratory function, but this effect may be influenced by publication bias. Overall, our results show that exposure to stressors during development negatively affects mitochondrial respiratory function, suggesting that changes in cellular metabolism may link developmental stressors to variation in whole animal traits and individual fitness.

## I. INTRODUCTION

### (1) Developmental stressors – phenotypic and fitness effects

The developmental environment has long been recognized as a powerful force that shapes adult phenotypes and fitness (Mousseau & Fox, 1998; Sheldon, 2002; Stamps, 2003; Lindstrom, 1999). In many taxa, the environment in which an individual develops can shape morphology, physiology, performance, and behaviour with consequences for reproductive success and survival (Noble, Stenhouse & Schwanz, 2018; Eyck *et al*., 2019; Lu *et al*., 2019; O'Dea *et al*., 2019; Buchanan *et al*., 2004; Crino & Breuner, 2015). Furthermore, the developmental conditions experienced in one generation can influence future generations through non‐genetic parental effects and heritable changes in gene expression related to histone methylation and other epigenetic modifications (transgenerational effects; Legoff *et al*., 2019; Ruuskanen, 2024). Overall, developmental conditions can affect individual fitness, which in turn alters population‐level dynamics and shapes ecological and evolutionary patterns (Love, McGowan & Sheriff, 2013; Monaghan, 2008; Chaby, 2016).

Developmental effects have been extensively studied in vertebrates in the context of developmental stressors (reviewed in Wada & Coutts, 2021; McGowan & Matthews, 2018; Nesan & Vijayan, 2013; Spencer & MacDougall‐Shackleton, 2011; Crino & Breuner, 2015). Although ‘developmental stressors’ is a broad term, it generally refers to a range of sub‐optimal conditions experienced by animals during pre‐ or postnatal development (Crino & Breuner, 2015). These conditions include energetic and nutritional imbalances such as excessive or restricted nutrition, exposure to elevated levels of glucocorticoids (steroid hormones that regulate metabolism and stress responses in vertebrates), psychological disturbances such as reduced social contact and anthropogenic stressors, and reduced parental care (reviewed in Crino & Breuner, 2015; Eyck *et al*., 2019; Spencer & MacDougall‐Shackleton, 2011). Developmental stressors affect many traits, including cognition, immune function, body size, morphology, animal performance, sexually selected traits, and life‐history strategies (e.g. Kraft *et al*., 2024; Brumm *et al*., 2021; Crino *et al*., 2024c; Crino *et al*., 2022). The effects of developmental stressors can persist across an individual's lifespan and affect future generations through parental effects and heritable epigenetic changes (reviewed in Vaiserman, 2015; Crino *et al*., 2024a). In these ways, developmental stressors experienced in one generation can affect individual survival and reproductive success in subsequent generations, ultimately altering longer‐term population‐level trends (Crino *et al*., 2024a; Mentesana & Hau, 2022; Constantinof, Moisiadis & Matthews, 2016).

Although the effects of developmental stressors on whole‐animal traits have been well studied, far less is known about the underlying cellular mechanisms that regulate such effects. Recently, mitochondrial respiratory function (see Section I.2) has been identified as an important cellular mechanism that links developmental conditions to both short‐ and long‐term changes in phenotype, performance, and pathology (reviewed in Zhao *et al*., 2022; Gyllenhammer *et al*., 2020; Daniels, Olsen & Tyrka, 2020). Here, we review pathways by which developmental stressors can modify mitochondrial respiratory function, potentially leading to sustained changes in organismal traits and individual fitness. Additionally, we use meta‐analysis to test the effects of four developmental stressors (glucocorticoid exposure, nutritional imbalance, parental care deprivation, and psychological disturbance) on mitochondrial respiratory function in multiple animal taxa. We evaluated how factors such as developmental stage (prenatal *versus* postnatal) and taxa influenced the general effects of developmental stressors on different aspects of mitochondrial respiratory function to understand the physiological trade‐offs operating at a cellular level that influence animal phenotype, performance, and fitness.

### (2) Fundamentals of mitochondrial biology and terminology

Mitochondria are cell organelles that play key roles in multiple cellular processes including calcium signalling, apoptosis, steroid synthesis, and the production of adenosine triphosphate (ATP) through the process of oxidative phosphorylation (OXPHOS; Melchinger & Garcia, 2023; Picard & Shirihai, 2022; Wang & Youle, 2009; Picard *et al*., 2018). ATP is also produced in the cytosol through glycolysis but in far lower quantities compared to ATP production by OXPHOS, earning mitochondria the moniker of ‘powerhouses of the cell’ (Siekevitz, 1957). Mitochondria vary in both the rate and efficiency at which they produce ATP which, in turn, affects the production of reactive oxygen species (ROS; Brand, 2005; Koch *et al*., 2021). ROS are normal byproducts of cellular respiration that can damage mitochondrial and cellular proteins, DNA, and lipids (Kuznetsov *et al*., 2022). Antioxidant enzymes such as catalase (CAT), superoxide dismutase (SOD), and glutathione peroxidase (GPx) neutralize ROS to prevent the accumulation of oxidative damage (Ighodaro & Akinloye, 2018; Nandi *et al*., 2019). However, when ROS production exceeds antioxidant capacity, oxidative damage increases, leading to oxidative stress and contributing to senescence (Kowaltowski & Vercesi, 1999; Palma *et al*., 2024; Metcalfe & Monaghan, 2001). Together, mitochondrial efficiency, ROS production, and antioxidant capacity interact to shape ATP availability and cellular redox balance, with cascading effects on organismal function and performance.

The language used to discuss the capacity and efficiency of mitochondria to generate ATP varies across research groups and fields. For example, in biomedical research, *mitochondrial dysfunction* is often discussed in terms of pathologic deficits in energy metabolism (e.g. Pieczenik & Neustadt, 2007; Lin & Beal, 2006; Kim, Wei & Sowers, 2008). In comparison, researchers in the fields of physiology, ecology, and evolution often use terms like *mitochondrial function*, *energy and oxidative metabolism*, *mitochondrial bioenergetics*, or *mitochondrial quality* that focus on the ability of mitochondria to produce ATP (e.g. Koch *et al*., 2021; Hill, 2014; Crino *et al*., 2024b). Other researchers advocate for the movement away from terms such as mitochondrial function and mitochondrial dysfunction and toward a hierarchical terminology system that categorizes mitochondrial traits across levels of biological organization (Monzel, Enriquez & Picard, 2023).

Here, we use the term *mitochondrial respiratory function* to refer to the capacity of mitochondria to efficiently generate ATP. According to this definition, mitochondrial and cellular traits that potentially increase ATP production have a positive effect on mitochondrial respiratory function, while traits that potentially decrease ATP production have a negative effect on mitochondrial respiratory function. Therefore, we consider elevated levels of antioxidants and traits associated with respiration that increase ATP production to be indicative of *high* mitochondrial respiratory function. In contrast, we consider elevated levels of ROS, oxidative damage, and respiration traits associated with inefficient production of ATP (e.g. elevated uncoupling protein expression, or leak respiration) to be indicative of *low* mitochondria respiratory function. We recognize that broadly categorizing mitochondrial and cellular traits as having a positive or negative effect on mitochondrial respiratory function minimizes both the complex interactions between cellular and mitochondrial traits and the role of mitochondrial plasticity in optimizing animals to environmental conditions (e.g. the ‘uncoupling to survive’ hypothesis; Brand, 2000). However, we argue it is necessary to make simplifying assumptions about the complexity of mitochondrial physiology to gain a broad understanding of the effects of developmental conditions on mitochondrial respiratory function.

### (3) Developmental stressors and mitochondrial respiratory function – evidence from studies on nutritional imbalance and glucocorticoid exposure

Mitochondrial respiratory function can vary plastically among tissues within an organism according to homeostatic state and life‐history stage; it also varies among individuals and across populations, even in the absence of genetic differences among individuals/populations (e.g. Salin *et al*., 2015; Cooper, Hammad & Montooth, 2014; Casagrande *et al*., 2023). Such plastic mitochondrial responses allow organisms to meet changing metabolic demands depending on environmental conditions and/or their life‐history stages (Hood, 2024; Heine & Hood, 2020; Hill & Hood, 2025). Although mitochondria are plastic, developmental conditions can canalize their function such that responses in later life are modulated by early life experiences (Fig. 1; Grilo *et al*., 2021; Gyllenhammer *et al*., 2020). These ‘programmatic’ changes to mitochondrial respiratory function have been proposed to underlie the long‐term effects of developmental conditions on phenotype (Gyllenhammer *et al*., 2020; Grilo *et al*., 2021; Fernandez‐Twinn & Ozanne, 2010; Symonds *et al*., 2009). Several observations support this hypothesis. First, developmentally induced changes in mitochondrial respiratory function can persist past early life into adulthood and can even be inherited by subsequent generations (e.g. Saben *et al*., 2016; Hanafi *et al*., 2016; Alfaradhi *et al*., 2014; Casagrande *et al*., 2020; Stier, Monaghan & Metcalfe, 2022). Second, developmental conditions often affect a range of traits within an individual, suggesting that these responses are influenced by cellular mechanisms that are shared across cell and tissue types (Gyllenhammer *et al*., 2020). Third, mitochondrial respiratory function is intrinsically linked to physiological stress responses in vertebrates because glucocorticoid hormones are produced by mitochondria and interact with them to regulate energy availability (Picard *et al*., 2018; Lee *et al*., 2013; Miller, 2011; Manoli *et al*., 2007). Together, these observations suggest mechanistic pathways by which programmatic changes in mitochondrial respiratory function link developmental conditions to sustained changes in phenotype and performance.

**Fig. 1:**
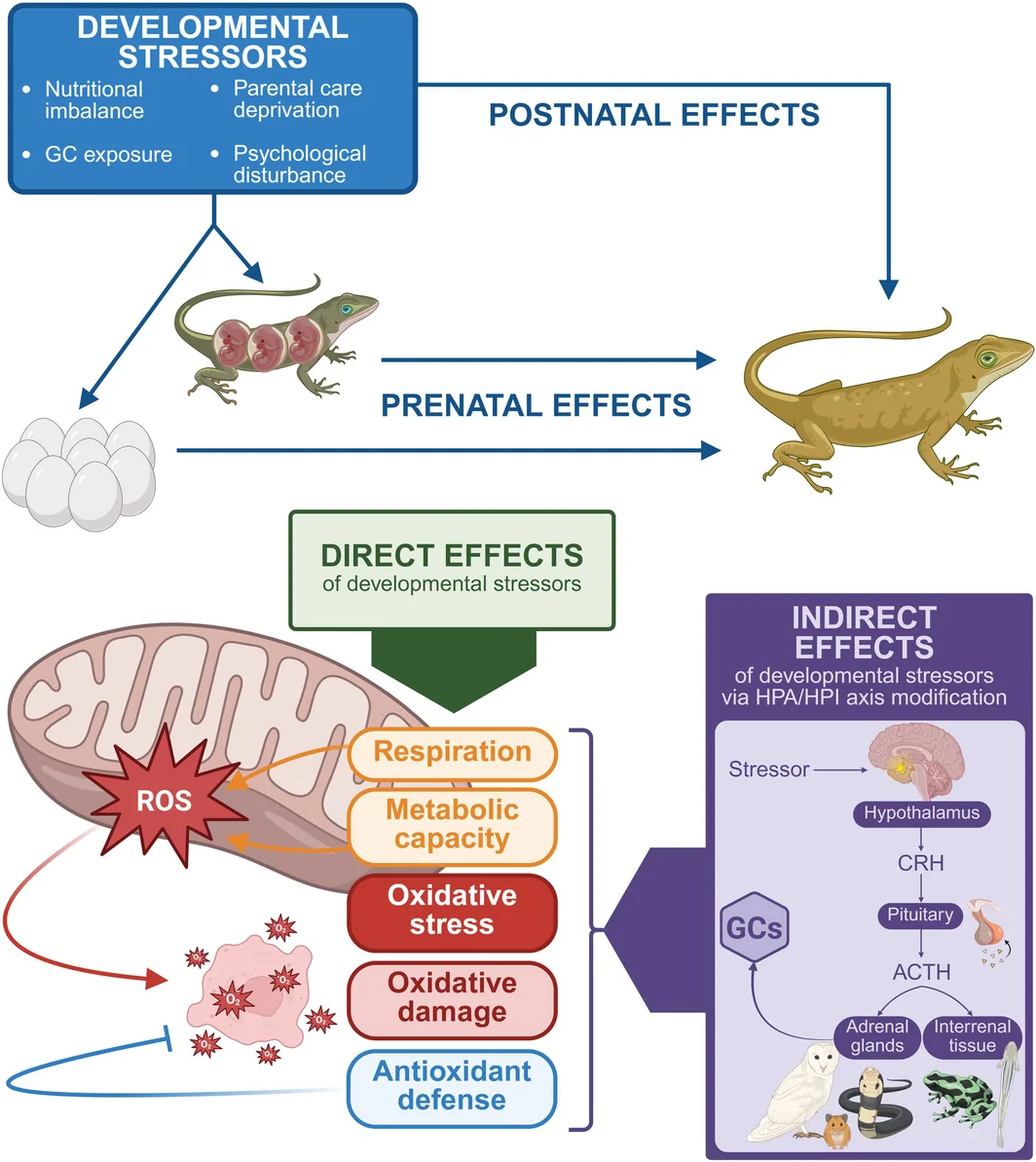
Pathways linking developmental stressors to mitochondrial respiratory function. Developing animals can be exposed to stressors both prenatally (during gestation or *in ovo*) and postnatally though direct exposure to environmental conditions and, in some species, though parental effects including parental care and maternal effects. The term ‘developmental stressors’ is broadly used to refer to conditions that are sub‐optimal such as exposure to elevated levels of glucocorticoid (GC) hormones, parental care deprivation, nutritional imbalance (i.e. excessive or insufficient nutrition), and psychological disturbances such as social isolation and exposure to anthropogenic disturbances such as artificial light and noise. Exposure to stressors during development can affect mitochondrial respiratory function through direct effects on traits that affect aerobic respiration, metabolic capacity, oxidative stress, oxidative damage, and antioxidant defences. Additionally, developmental stressors can indirectly affect mitochondrial respiratory function through programmatic effects on the hypothalamic–pituitary–adrenal/interrenal (HPA/HPI) axis. In vertebrates, the HPA/I axis regulates the release of GCs that play important roles in metabolic responses to energetically demanding or stressful conditions. Exposure to stressors during development can have sustained effects on HPA/I axis function across life and, in this way, indirectly affect mitochondrial respiratory function through changes in GC secretion and regulation. ACTH = adrenocorticotropic hormone; CRH = corticotropin‐releasing hormone; ROS = reactive oxygen species. Created in BioRender. Gardner, M. (2026) https://BioRender.com/sirsv2t is licensed under CC BY 4.0.

A substantial body of biomedical research has investigated the links between nutritional imbalances during development, mitochondrial respiratory function, and the emergence of metabolic disorders, cancer, and other pathologies later in life (reviewed in Grilo *et al*., 2021; Symonds *et al*., 2009; Hoffman *et al*., 2021). At all life stages, nutritional imbalances such as macronutrient excess or deficiency affect mitochondrial traits including biogenesis, structure, antioxidant biosynthesis, and OXPHOS activity (Kereliuk, Brawerman & Dolinsky, 2017; Armitage *et al*., 2004). During development, the nutritional environment can program physiological systems through changes in mitochondrial respiratory function that persist throughout life and can even be inherited by subsequent generations (Burdge *et al*., 2007; Grilo *et al*., 2021). In scenarios where the developmental environment matches the post‐developmental environment, programmatic developmental effects can induce physiological changes that enhance the likelihood of survival and thus potentially fitness (i.e. the ‘thrifty phenotype’ hypothesis; Hales & Barker, 1992). Conversely, mismatches between the developmental and post‐developmental environments, such as restricted nutrition during development and excessive nutrition during the postnatal period, can induce physiological changes that increase the likelihood of metabolic dysfunction and disease and early mortality (Gluckman & Hanson, 2004; Wells, 2003, 2006). Due to the links between mitochondrial respiratory function and human health and metabolic diseases, numerous studies have examined the effects of early life nutrition on mitochondrial respiratory function. However, it is still unclear which macronutrients (fat, carbohydrates, protein) are most influential in driving the effects of nutrition on mitochondria function (Kereliuk *et al*., 2017; Rodríguez‐Cano *et al*., 2020). Additionally, many studies in medical sciences use laboratory mice and rats as model systems to investigate how nutritional imbalances affect mitochondrial respiratory function and metabolic disease. The use of laboratory mice and rats as model systems may limit our ability to extrapolate findings to the genetically diverse, ecologically variable contexts that shape evolutionary processes. For this reason, it is unclear whether effects from laboratory rodents are representative of how mitochondria respond in other animal taxa (Milani & Ghiselli, 2020).

Studies examining the effects of glucocorticoid exposure during development have also provided empirical support linking developmental conditions to mitochondrial respiratory function. Developing animals can be exposed to glucocorticoids from maternal sources during gestation *in ovo*, and postnatally from breast milk in mammals and from their own endogenous production in response to postnatal disturbances (Groothuis *et al*., 2019; Stead, Badescu & Boonstra, 2022; Moisiadis & Matthews, 2014). Glucocorticoids (GCs) interact with glucocorticoid receptors (GRs) and mineralocorticoid receptors (MRs) to regulate mitochondrial and nuclear‐encoded genes that affect mitochondrial biogenesis, structure, and the cellular machinery that underlies production of ATP and ROS (Kokkinopoulou & Moutsatsou, 2021; Lee *et al*., 2013). In developing animals, such direct changes in mitochondrial respiratory function could have both short‐term and/or sustained effects on growth, development, immune function, and other traits. Additionally, exposure to glucocorticoids during development could indirectly affect mitochondrial respiratory function through programmatic effects on the neuroendocrine pathway that regulates the production of glucocorticoids (the hypothalamic–pituitary–adrenal/interrenal axis, HPA/I axis). Exposure to glucocorticoids during development can induce epigenetic changes to genes that reduce the expression of mRNA and proteins associated with GR levels (Jimeno *et al*., 2019). Such epigenetic effects have been linked to lifelong changes to the HPA/I‐axis that affect glucocorticoid regulation (reviewed in Matthews & McGowan, 2019; Buschdorf & Meaney, 2016; Grace, Kim & Rogers, 2011; Meaney, Szyf & Seckl, 2007). In this way, exposure to elevated glucocorticoids during development can indirectly influence mitochondrial respiratory function through sustained changes in endogenous levels of glucocorticoids (Crino *et al*., 2022). This indirect pathway highlights a potential mechanism to explain how multiple types of developmental stressors can result in both acute and sustained effects on mitochondrial respiratory function that, in turn, affect the expression of phenotypic traits and performance (Picard & McEwen, 2018).

### (4) Assessing the effects of developmental stressors on mitochondrial respiratory function – a meta‐analytical approach

Despite the large body of research examining the sustained effects of developmental stressors on mitochondrial respiratory function, it is unclear if these effects are widespread across taxonomic groups. It is also unclear which components of mitochondrial respiratory function are most likely to be affected. The effects of developmental stressors on mitochondrial traits are likely to be impacted by the type of stressor (e.g. nutritional imbalance, glucocorticoid exposure), the timing of exposure to the stressor (i.e. prenatal or postnatal), the duration and magnitude of stressors, the sex of individuals subjected to the stressor, and taxon to which the subjects belong. Furthermore, the persistence of developmentally induced changes to mitochondrial respiratory function across an animal's life is likely to be similarly dependent on context and could also vary based on the environmental conditions that animals experience following development.

To provide answers to some of these questions, we conduct a meta‐analysis to test how developmental stressors affect mitochondrial traits related to aerobic respiration using experimental studies in multiple animal taxa. We evaluated the effects of four developmental conditions that are commonly manipulated in experiments that test phenotypic responses to developmental stressors on mitochondrial respiratory function, namely: glucocorticoid exposure, nutritional imbalance, parental care deprivation, and psychological disturbance such as handling stress, light exposure, and social isolation (Table 1). Our main objective was to uncover the broad effects of developmental stressors on a range of mitochondrial and cellular traits that relate to mitochondrial respiratory function including the capacity and efficiency of cellular respiration, antioxidants, oxidative stress, and oxidative damage (Table 2). Additionally, we sought to understand how factors such as the type of developmental stressor, timing of exposure to stressors, sex, and taxa influence the magnitude, direction, and duration of the effects of developmental stressors on mitochondrial respiratory function.

**Table 1:** Categories of conditions that are commonly used to manipulate development stress in vertebrates.

Category	Description of manipulation	Examples	References
Glucocorticoid exposure	Increased exposure to glucocorticoid hormones either directly through exposure of study animal to elevated levels or indirectly through maternal exposure	Glucocorticoid supplementation through feeding, injection of glucocorticoids into eggs, maternal exposure through dexamethasone injection	Casagrande *et al*. (2020), Chen *et al*. (2021), Cossin‐Sevrin *et al*. (2022), Crino *et al*. (2022), Recio *et al*. (2025a)
Nutritional imbalance	Manipulations that alter total calories or macronutrients including carbohydrates, fat, and protein (can include *Overnutrition* and *Undernutrition*), can be indirect (imposed on mothers) or direct (imposed on study animal)	Diets with an overabundance of calories or restricted calories, high/low macronutrient diets, clutch/litter size manipulations that affect food availability	Braz *et al*. (2020b), Castro *et al*. (2015), Jousse *et al*. (2014), Santana *et al*. (2019)
Parental care deprivation	Removal of parent (often mother) during postnatal care	Rodent mothers removed for period during postnatal care (e.g. 3 h/day)	Abelaira *et al*. (2021), Banqueri *et al*. (2021), do Prado *et al*. (2016)
Psychological disturbance	Exposure to stimuli that are considered stressful and could increase glucocorticoid levels (although not necessarily measured)	Handling stress, light exposure, maternal restraint during gestation, tank water draining or agitation, social isolation	Castro *et al*. (2018), Sonei *et al*. (2017), Toniazzo *et al*. (2019), Zheng *et al*. (2015)

**Table 2:** Categories of mitochondrial and cellular traits that affect mitochondrial respiratory function.

Category	Description/action	Examples	References
**Antioxidant defence**	Molecules that directly neutralize reactive oxygen species (i.e. antioxidants) or indirectly contribute to antioxidant defence by supplying electrons to drive redox reactions	Catalase (CAT), reduced glutathione (GSH), superoxide dismutase (SOD)	Napolitano *et al*. (2021), Oyewole & Birch‐Machin (2015)
* Enzymatic*	Catalyze reactions that neutralize reactive oxygen species (ROS) and reactive nitrogen species (RNS)	CAT, glutathione peroxidase (GPx), glutathione reductase (GR), glutathione S‐transferase (GST), SOD	Abelaira *et al*. (2021), Madkour *et al*. (2023), Prokic *et al*. (2021)
* Non‐Enzymatic*	Neutralize ROS and RNS by donating electrons	GSH, sulfhydryls, thiols, total radical‐trapping antioxidant potential (TRAP)	Amini‐Khoei *et al*. (2017), Prokic *et al*. (2021), Stone *et al*. (2016)
* General*	Total overall capacity of a biological sample to neutralize free radicals	Total antioxidant capacity (TAC or T‐AOC), total antioxidant response (TAR), NADPH	Baker *et al*. (2007), Ferreira *et al*. (2016b), Wang *et al*. (2019)
**Metabolic capacity**	The scope of mitochondria to produce ATP	ATP, β‐hydroxyacyl‐CoA dehydrogenase (HOAD), citrate synthase, cytochrome c oxidase (COX), metabolic enzymes (e.g. aspartate aminotransferase), electron transport system (ETS) protein complexes (complex I–V), membrane potential, mitochondrial number/density, mitochondrial traits (e.g. cristae length, mitochondrial swelling)	Banqueri *et al*. (2021), Burness *et al*. (2000), Simoes‐Alves *et al*. (2019), Wen *et al*. (2017)
**Oxidative damage**	Changes to the structure of molecules due to reactive oxygen species (ROS) and reactive nitrogen species (RNS)	Malondialdehyde (MDA), protein carbonyls	Brand & Nicholls (2011), Nissanka & Moraes (2018)
* DNA*	Oxidative damage of DNA	8‐hydroxy‐2'‐deoxyguanosine (8‐OHdG), apurinic/apyrimidinic (AP) sites in genomic DNA	Dettleff *et al*. (2020), Reichert *et al*. (2015), Stier *et al*. (2020)
* Lipids*	Oxidative damage of lipids	4‐hydroxynonenal (4‐HNE) adducts, MDA, thiobarbituric acid reactive substances (TBARS)	Prokic *et al*. (2021), Zengin (2021), Zhang *et al*. (2009)
* Proteins*	Oxidative damage of proteins	3‐nitrotyrosine (3NT), protein carbonyls	Alfaradhi *et al*. (2014), Feng *et al*. (2012), Réus *et al*. (2017)
* General*	Measures that capture oxidative damage across a range of molecules	Reactive oxygen metabolites (ROM), global oxidative status (oxy‐score)	Braz *et al*. (2020b), Costantini (2010)
**Oxidative/nitrosative stress**	An imbalance between reactive oxygen/nitrogen species production and antioxidant defences	Hydrogen peroxide (H_2_O_2_), nitrite, GSH/oxidized GSH (GSSG), superoxide anion (e.g. ROS)	Abelaira *et al*. (2021), Recio *et al*. (2025b), do Prado *et al*. (2016), Yap *et al*. (2022)
**Respiration**	Mitochondrial oxygen consumption	Basal respiration, oxidative phosphorylation (OXPHOS) respiration	Brand (2005), Brand & Nicholls (2011)
* Basal*	Oxygen consumed in the presence of metabolic substrates but without exogenous ADP stimulation	Cellular metabolic rate, state 2 respiration	de Moura Freitas *et al*. (2019), Gallagher *et al*. (2005), Price *et al*. (2017)
* Total OXPHOS*	Total oxygen consumed during oxidative phosphorylation	ADP‐stimulated respiration, maximal respiration, OXPHOS respiration, routine, state 3	Casagrande *et al*. (2020), Ferreira *et al*. (2016a), Price *et al*. (2017)
* ATP‐linked OXPHOS*	Oxygen consumption linked to the production of ATP during oxidative phosphorylation (= *Total OXPHOS* − *Leak*)	OXPHOS respiration	Cossin‐Sevrin *et al*. (2022), Crino *et al*. (2022), Stier *et al*. (2022)
* Leak*	Oxygen consumed due to proton leak; often measured using oligomycin to inhibit ATP synthase	State 4 respiration, leak respiration, non‐phosphorylating respiration	Crino *et al*. (2022), de Moura Freitas *et al*. (2019), Khamoui *et al*. (2018)
* ETS*	Oxygen consumed when the ETS is uncoupled from ATP synthesis	Maximum uncoupled respiration	Braz *et al*. (2020b), Casagrande *et al*. (2020), Yap *et al*. (2022)
* Mitochondrial coupling*	Measures of how efficiently mitochondria use oxygen and substrates to support ATP production	ATP yield per oxygen molecule consumed (ATP/O), Coupling efficiency, flux control ratios (FCR_L/R_, FCR_L/ETS_), OXPHOS coupling efficiency (OxCE), respiratory control ratio (RCR), phosphate‐to‐oxygen (P/O) ratio	Jorgensen *et al*. (2009), Stier *et al*. (2022), Voituron *et al*. (2022)
* Mitochondrial capacity*	Measures of the fraction of ETS maximum capacity used under endogenous cellular conditions	FCR_R/ETS_, mitochondrial aerobic scope (ETS – leak)	Cossin‐Sevrin *et al*. (2022), Stier *et al*. (2022), Voituron *et al*. (2022)

Uncovering the general effects of developmental stressors on mitochondrial respiratory function will illuminate the physiological trade‐offs operating at a cellular level that influence animal phenotype and performance. Mitochondrial respiratory function is not only integral to organismal performance but also underpins many evolutionary and ecological processes. Understanding how the developmental environment shapes mitochondrial respiratory function across life will provide insight into broad ecological and evolutionary patterns, such as how organisms acclimate to novel environments, variation in the expression of sexually selected traits, and speciation (Koch *et al*., 2021; Koch & Hill, 2018). Additionally, uncovering the general effects of developmental stressors on mitochondrial respiratory function will enable us to generate testable hypotheses and direct future research to fill gaps in our foundational knowledge. Finally, mitochondrial respiratory function is linked to a wide range of pathologies and diseases in humans including neurodegenerative and metabolic disorders, cancer, and autoimmune diseases (Lin & Beal, 2006; Boland, Chourasia & Macleod, 2013; Gómez‐Serrano *et al*., 2018; Pieczenik & Neustadt, 2007). A deeper understanding of the interaction between developmental conditions and mitochondria function may help inform early intervention strategies and targeted therapies in human medicine.

## II. METHODS

### (1) Data collection

We conducted a systematic literature search for published studies that tested the effects of developmental stressors on mitochondrial respiratory function following the Preferred Reporting Items for Systematic Reviews and Meta‐Analyses (PRISMA) and the PRISMA‐EcoEvo guidelines (O'Dea *et al*., 2021). We developed a string of search terms aimed at capturing studies that administered stress treatments (including glucocorticoid hormones, nutritional imbalance, parental care deprivation, and psychological disturbance) during development and measured mitochondrial respiratory function (e.g. oxygen consumption, mitochondrial biogenesis, oxidative damage, ROS production; see Appendix S1 for full search string). Our initial search string also included terminology to capture studies that manipulated temperature during development as an additional developmental stressor category. However, after screening studies and collecting data but prior to data analysis, we elected to remove studies that manipulated temperature. This was done with the intention that this data be used in a separate meta‐analysis. We searched the Scopus and Web of Science (v.5.35) databases using our search string on the 31st of October 2022. Our searches returned 5,293 peer‐reviewed articles using Web of Science and 1,996 using Scopus. Of the initial 7,289 articles, 362 were duplicated across searches and were removed, resulting in 6,927 studies that were subjected to further screening (Fig. 2).

**Fig. 2:**
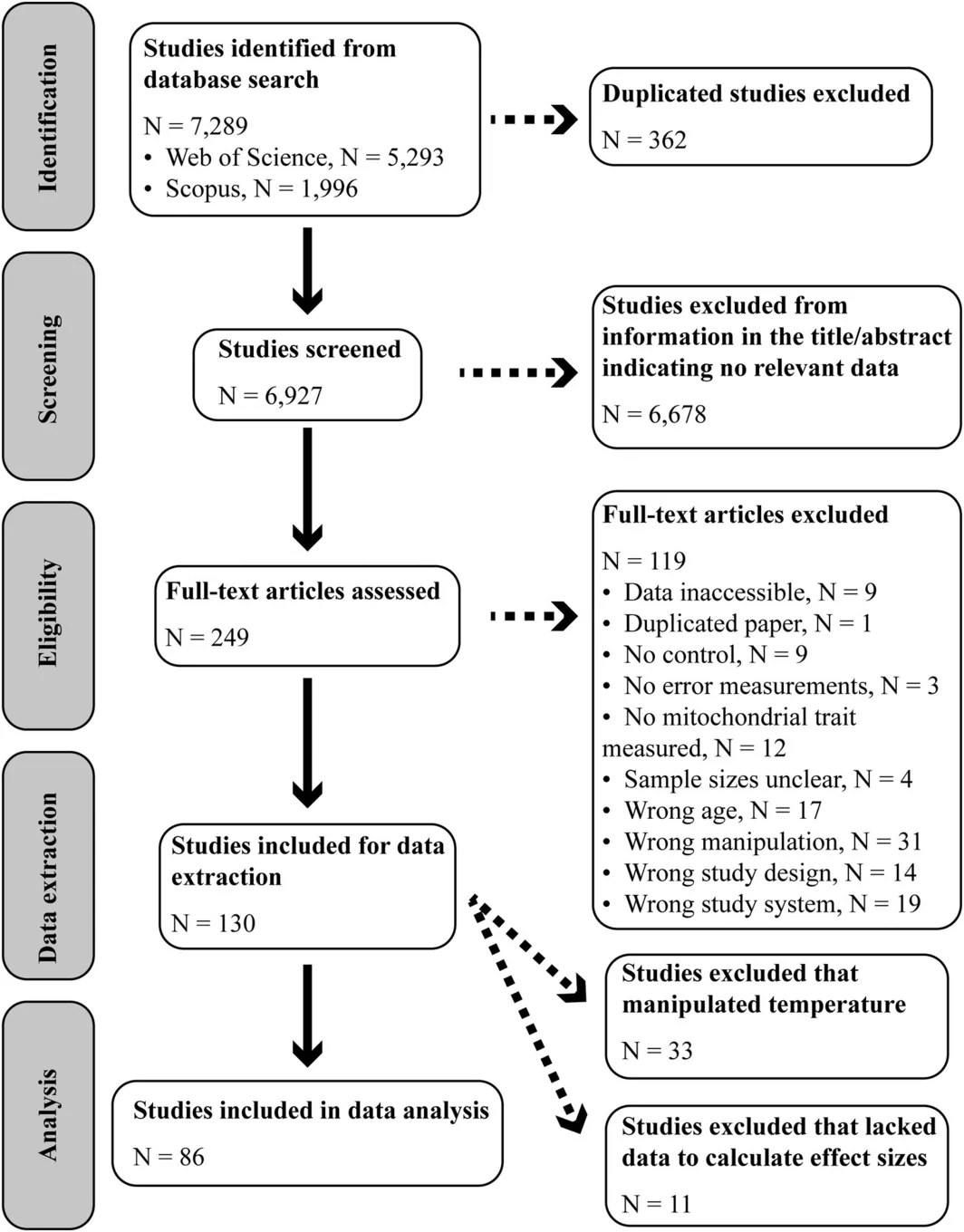
Flowchart of search results and study selection.

We (OC, GH, DL, DN, PR, KW) screened titles and abstracts using Rayyan, a web application for systematic reviews (Ouzzani *et al*., 2016). We evaluated whether articles presented results from experiments where developmental conditions were manipulated during the pre‐ and/or postnatal periods and measured mitochondrial parameters associated with antioxidants, cellular respiration, metabolic capacity, oxidative damage, or oxidative stress (Table 2). We included studies that measured mitochondrial respiratory function from isolated mitochondria, cells, or plasma/serum (capturing systematic effects of mitochondrial respiratory function). We defined the postnatal period from hatching or birth to sexual maturity and thus included studies that manipulated conditions during the juvenile period. We focused on studies that manipulated developmental conditions through exposure to stressors including glucocorticoid exposure, parental care deprivation, nutritional imbalance, or exposure to psychological disturbances (e.g. light, social isolation; Table 1). To be included, studies had to meet the following criteria: (*i*) manipulate exposure to a stressor, (*ii*) be conducted on animals prior to sexual maturity, and (*iii*) measure a cellular or mitochondrial trait linked to mitochondrial respiratory function (Table 2). We excluded studies that were conducted using cell culture, exposed all experimental animals to multiple developmental manipulations (e.g. temperature and nutrition), and studies that treated animals with drugs or nutritional supplements in addition to stressors. Additionally, we excluded studies where animals were exposed to stressors for a period that overlapped development and sexual maturity. Using these criteria, we excluded all but 249 studies. We read these papers in their entirety and excluded a further 119 studies for the following reasons: data inaccessible (*N* = 9), duplicated paper (*N* = 1), no control group (*N* = 9), no error measurements (*N* = 3), no mitochondrial trait measured (*N* = 12), wrong age (*N* = 17), sample sizes unclear (*N =* 4), wrong manipulation (*N* = 31), wrong study design (e.g. treatments included multiple developmental stressors, *N* = 14), and wrong study system (e.g. treatments were applied after animals reached sexual maturity, *N* = 19). We excluded a further 33 studies that manipulated temperature during development for use in a separate meta‐analysis, resulting in 97 studies for data extraction. We (OC, DL, DN, KW) extracted means, standard deviations, and sample sizes from text, tables and figures. Data from figures were digitized using the *metaDigitise* R package (Pick, Nakagawa & Noble, 2019). In all cases, we used the number of unique individuals in each treatment group as the replicates for determining sample size. Following initial data extraction, we excluded 11 studies because of uncertainty about the data needed to calculate effect sizes, which could not be resolved through correspondence with authors, resulting in 86 studies included in the final analysis (Fig. 2, Appendix S2).

### (2) Calculating effect sizes

To quantify the impacts of developmental stress on mitochondrial respiratory function we calculated the standardized mean difference between measurements from treatment and control groups, assuming heteroscedastic population variances (SMD_H_) (Bonett, 2008, 2009), along with its associated sampling error. We used the *escalc* function of the *metafor* package (vers. 4.8.0; Viechtbauer, 2010) in R (vers. 4.5.1) which corrects for possible bias resulting from effect sizes calculated with small sample sizes (Borenstein & Hedges, 2009). We choose to use SMD_H_ as our effect size because our data contained: (*i*) substantial amounts of ratio data (e.g. respiratory control ratios, relative gene expression), (*ii*) percentages and zero measurement variables, and (*iii*) skewed measurement variables. SMD_H_ is more robust to these types of measurement variables compared to alternative effect size measures, such as the log response ratio.

We calculated SMD_H_ as the mean difference between control and treatment groups divided by the pooled standard deviation. As such, positive effect sizes represent outcomes where the mean of the experimental group was larger than the control group. Given the diversity of measurement variables, we paid particular attention to effect size direction to ensure that positive effects were consistent with our definition of increased mitochondrial respiratory function. If necessary, we corrected the direction of effect size (multiplied by −1) to ensure their interpretation remained consistent with respect to mitochondrial respiratory function. Effects were excluded if sample sizes or standard deviations were missing.

### (3) Meta‐analysis

We analysed our data using multilevel meta‐analytic (MLMA) and meta‐regression (MLMR) models in R using the *rma*.*mv* function in *metafor* (vers. 4.8.0; Viechtbauer, 2010). Meta‐analytic mean estimates and meta‐regression models were plotted using orchard plots which convey overall meta‐analytic means, alongside raw effect sizes and prediction intervals, which describe effect heterogeneity using the *orchaRd* package (vers. 2.0; Nakagawa *et al*., 2021; Nakagawa *et al*., 2023). For all inferential tests, we consider mean effects to be significantly different from zero when confidence intervals did not overlap zero and/or when *P* < 0.05.

#### (a) Multi‐level meta‐analysis (MLMA) models

Using data subsets for each of our major stressor categories, we first fit MLMA models (i.e. intercept‐only models) with SMD_H_ that included study, biological matrix (i.e. the material from which mitochondrial parameters were quantified such as tissue type, plasma, or serum), and phylogeny as random effects to account for non‐independence and identify sources of variability. We also explored models that included species and sample dependency as random effects, but these were highly confounded with phylogeny and study, so we simplified models by removing these terms. We included an observation‐level random effect in our models because *metafor* does not estimate a residual variance by default. The size of datasets varied in the number of species and biological matrices used to measure mitochondrial traits (Table 3). As such, we simplified the random effect structure for some datasets to include only random effects with six or more levels. Our MLMA models allowed us to partition variation in SMD_H_ among these key sources while accounting for total sampling variance. We took a pluralistic approach to heterogeneity reporting, by reporting absolute ($\sigma^{2}$), relative (i.e. $I_{\text{total}}^{2}$, *sensu* Nakagawa & Santos, 2012; Noble *et al*., 2022), and magnitude measures (both $CV_{\text{total}}$ and $M_{\text{total}}$) of heterogeneity as suggested by Yang *et al*. (2025). We also report each of their stratified versions for each random effect level (Nakagawa & Santos, 2012; Yang *et al*., 2025). We present 95% prediction intervals which describe the expected distribution of effects from future studies (Nakagawa *et al*., 2021; Noble *et al*., 2022).

**Table 3:** Relative heterogeneity estimates ($I^{2}$), sample sizes ($n_{\text{effects}}$), number of studies ($n_{\text{study}}$), and number of species ($n_{\text{species}}$) for each of the developmental stressor categories. Relative heterogeneity is reported as $I^{2}$ (in percent) based on the intercept‐only multi‐level meta‐analytic model. Total heterogeneity, along with stratified version of $I^{2}$ (study, phylogeny, biological matrix, and observation) are also provided. Additional heterogeneity measures are provided in the Supporting Information (Tables S1 and S2).

Developmental stressor	$n_{\text{effects}}$	$n_{\text{species}}$	$n_{\text{study}}$	$I_{\text{total}}^{2}\left(\%\right)$	$I_{\text{study}}^{2}\left(\%\right)$	$I_{\text{phylogeny}}^{2}\left(\%\right)$	$I_{\text{matrix}}^{2}\left(\%\right)$	$I_{\mathrm{obs}}^{2}\left(\%\right)$
Glucocorticoid exposure	140	8	10	86	12.2	0	68	6.6
Psychological disturbance	190	8	16	80	57.2	11	0	11.8
Nutritional imbalance	645	15	51	80	8.6	13	10	47.9
Parental care deprivation	172	2	10	82	36.8	–	–	45.1

A phylogeny was derived by first cross‐checking taxa names using the Open Tree of Life (OTL) with the *rotl* package in R (vers. 3.1.0; Michonneau, Brown & Winter, 2016), and plotting taxa using *ggtree* to visualize the tree (vers. 3.16.0; Fig. 3; Yu *et al*., 2017). We resolved any taxa names that were outdated or changed. Once names in the dataset and *rotl* database matched, we exported species names and built a time calibrated phylogeny for the species in our dataset using TimeTree (timetree.org; Kumar *et al*., 2022). We used the R packages *ape* (vers. 5.8.1; Paradis & Schliep, 2019) and *phytools* (Revell, 2012) to prune the tree for individual analyses and calculate phylogenetic correlation matrices used in meta‐analytic models.

#### (b) Multi‐level meta‐regression (MLMR) models

After quantifying levels of heterogeneity in each of the different stressor datasets, we fit a series of multi‐level meta‐regression (MLMR) models to test our key questions. In all models, we included the same random effects as we used in our MLMA models. Several moderators were relevant to all stressor manipulation types including: (*i*) measurement trait category (e.g. antioxidants, oxidative damage, etc.), (*ii*) stage of manipulation (prenatal, postnatal, or both) and (*iii*) taxa (e.g. class: mammals, birds, amphibians, fish). Some stressor categories included studies that were only conducted in a single taxonomic class (e.g. parental care deprivation was only done in mammals) and so we did not include the taxa moderator in these models. We fit models assuming heteroscedastic variation among levels of a given moderator to capture differences in effect variation within groups.

We had a substantially larger dataset of studies measuring nutritional stress (*N =* 51 studies) compared to other categories of developmental stressors. These studies were more variable and so we included some additional moderators to capture variation in effects. These included: (*i*) whether the manipulation involved a nutrition surplus (i.e. overnutrition) or deficit (i.e. undernutrition), and (*ii*) the type of nutrition manipulation done (fat, protein, carbohydrate, or total food). We expected that different mitochondrial traits would respond differently to nutritional stress and so we also included an interaction between mitochondrial trait category and the type of nutrition manipulation (i.e. under‐ or overnutrition). However, we did not have enough data to estimate an interaction between the type of nutrition manipulation and the category of mitochondrial traits (e.g. antioxidants, oxidative stress, etc.). We fit three models that included: (*i*) only nutrition type as a moderator, (*ii*) only under/overnutrition as a moderator and (*iii*) a combined model with both main effects. We evaluated support among models using Akaike information criterion (AIC) and chose the simplest model (fewest estimated parameters) when the ΔAIC values were within two units of each other.

#### (c) Sensitivity analysis and publication bias

We checked for publication bias by first visually exploring plots between each effect size and its precision (1/SE) for each stressor type using funnel plots. Importantly, funnel asymmetry can be caused by non‐independence and high heterogeneity. As such, we tested for publication bias more formally by altering our overall MLMA models to include the square root inverse effective sample size as a moderator (Nakagawa *et al*., 2022). We used the inverse effective sample size because the sampling variance of SMD_H_ includes the effect size itself in its calculation inducing a correlation (Nakagawa *et al*., 2022). This method allows one to test for publication bias inferentially. When the slope of the moderator is significantly different from zero, it indicates that publication bias is potentially present. If the slope was significant, we refit models with the inverse effective sample size (as opposed to the square root inverse effective sample size) because this provides an unbiased estimate of the meta‐analytic mean (intercept; Nakagawa *et al*., 2022). Of course, we cannot determine if publication bias is truly present, so these analyses should be viewed as a sensitivity analysis to check the robustness of our results.

## III. RESULTS

In total, we collected 1147 effect sizes from 21 species (Fig. 3) and 86 studies across the four categories of developmental stressors (Table 3). There were clear taxonomic biases because most effects came from studies on four species of mammals (effects = 951, studies = 64; *Mus musculus*, *Rattus norvegicus*, *Ovis aries* and *Capra hircus*). Fish had the second most effects (105, species = 9 and studies = 10) followed by birds (75, species = 6 and studies = 11), and amphibians (16, species = 2 from only 1 study).

**Fig. 3:**
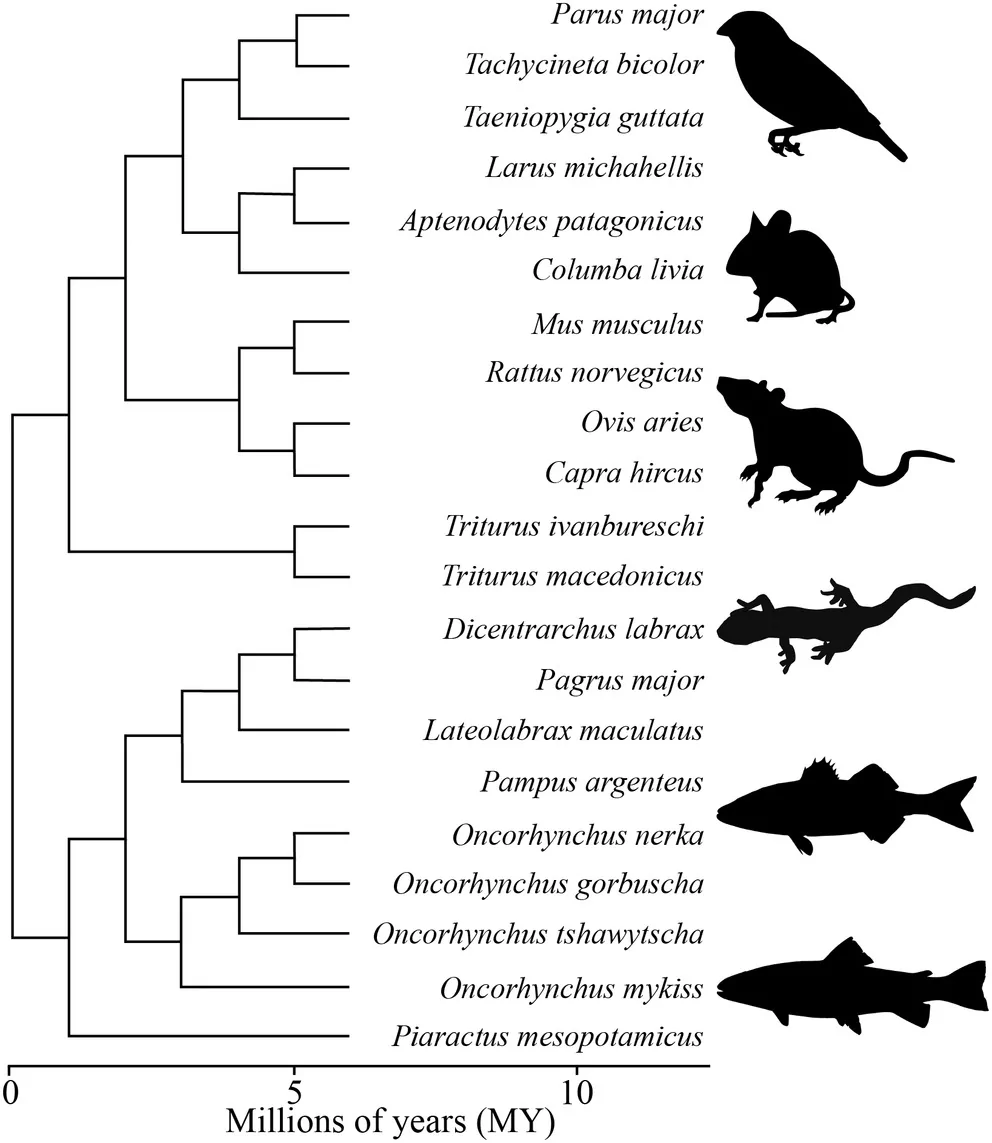
Phylogeny of species included in the meta‐analysis. Phylogenetic relationships and divergence times are based on TimeTree [timetree.org; Kumar *et al*. (2022)]. The phylogeny was pruned to only include species in the dataset. The animal silhouettes are from PhyloPic (www.phylopic.org) and were contributed by Andy Wilson (*Taeniopygia guttata*, 2023; CC0 1.0), Mozillian (*Mus musculus*, 2024; CC0 1.0), Beth Reinke (*Triturus* spp., 2021; CC0 1.0), Denis Lafage (2020; CC0 1.0), Steven Traver (*Oncorynchus mykiss*, 2012; CC0 1.0), Lihao (*Lateolabrax maculatus*; 2024; CC0 1.0), and Katy Lawler (*Rattus* spp., 2023; CC0 1.0).

### (1) Glucocorticoid exposure – impacts on mitochondrial respiratory function

Overall, exposure to glucocorticoids during development negatively affected mitochondrial respiratory function (SMD_H_ = −0.92, 95% CI = −1.69 to −0.16, P = 0.02; Fig. 4A). However, the effects of developmental stressors on mitochondrial respiratory function differed across contexts and studies. Specifically, effect heterogeneity was high (95% Prediction Intervals: −3.07 to 1.23; Table 3, see Tables S1 and S2), with 12.24% of variation being driven by differences among studies ($I_{\text{study}}^{2}$; Table 3) and 67.53% of variation being driven by differences among biological matrices ($I_{\text{matrix}}^{2}$; Table 3). However, little to no variation was explained by phylogeny (Table 3). We found no strong evidence that suggested the results were driven by publication bias (See Supporting Information, Fig. S1, Appendix S3).

**Fig. 4:**
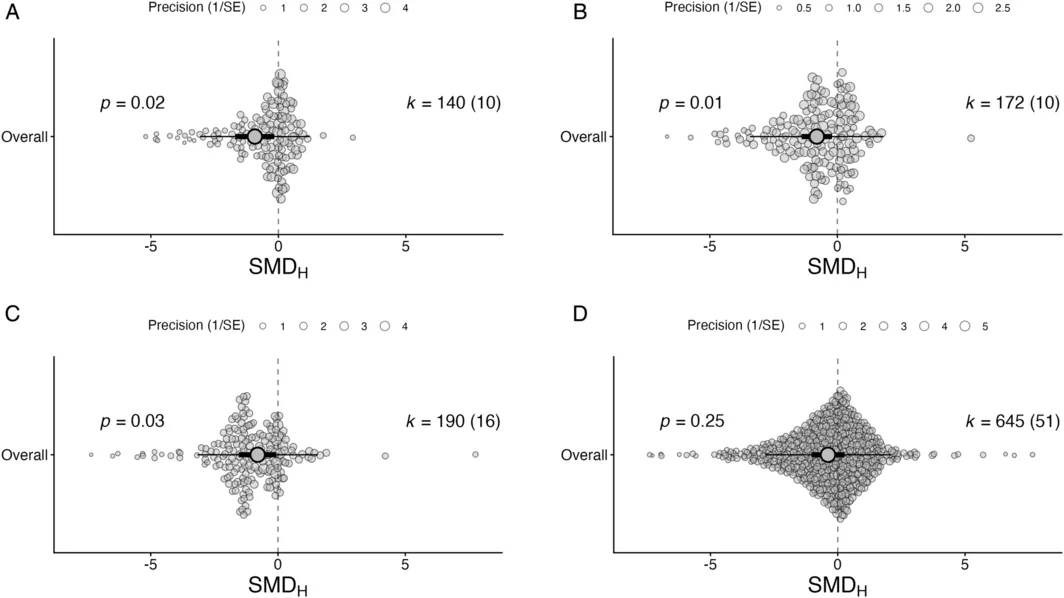
Overall meta‐analytic mean estimates for each developmental stressor category (A) glucocorticoid exposure, (B) parental care deprivation, (C) psychological disturbance, and (D) nutritional imbalance. Mean estimates are shown as large points, with 95% confidence (thick black bars) and prediction (thin black bars). The number of effects (*k*) along with the number of studies (in parentheses) are provided for each stressor category. The dashed lines represent no effect where the estimate of the standardized mean difference between the treatment and control groups (SMD_H_) equals zero. Raw data (translucent grey bubbles) are also shown and are weighted by their inverse sampling error (precision). Raw data is truncated for ease of visualisation. Note that there was some evidence for publication bias for studies on nutritional imbalance and psychological disturbance (see Section III).

Exposure to glucocorticoids during development decreased mitochondrial respiratory function across all mitochondrial trait categories with no significant differences between categories ($Q_{M_{4,135}}$ = 1.33, *P* = 0.26; Fig. 5A). However, glucocorticoids had the greatest negative impact on metabolic capacity (SMD_H_ = −1.1, 95% CI: −1.87 to −0.32, P = 0.01) and mitochondrial respiration (SMD_H_ = −0.81, 95% CI: −1.6 to −0.04, P = 0.04). Additionally, treatment with glucocorticoids during development increased oxidative damage (SMD_H_ = −0.67, 95% CI: −1.46 to 0.12, P = 0.1) and oxidative stress (SMD_H_ = −0.80, 95% CI: −1.73 to 0.13, P = 0.09), although these effects were not significant.

**Fig. 5:**
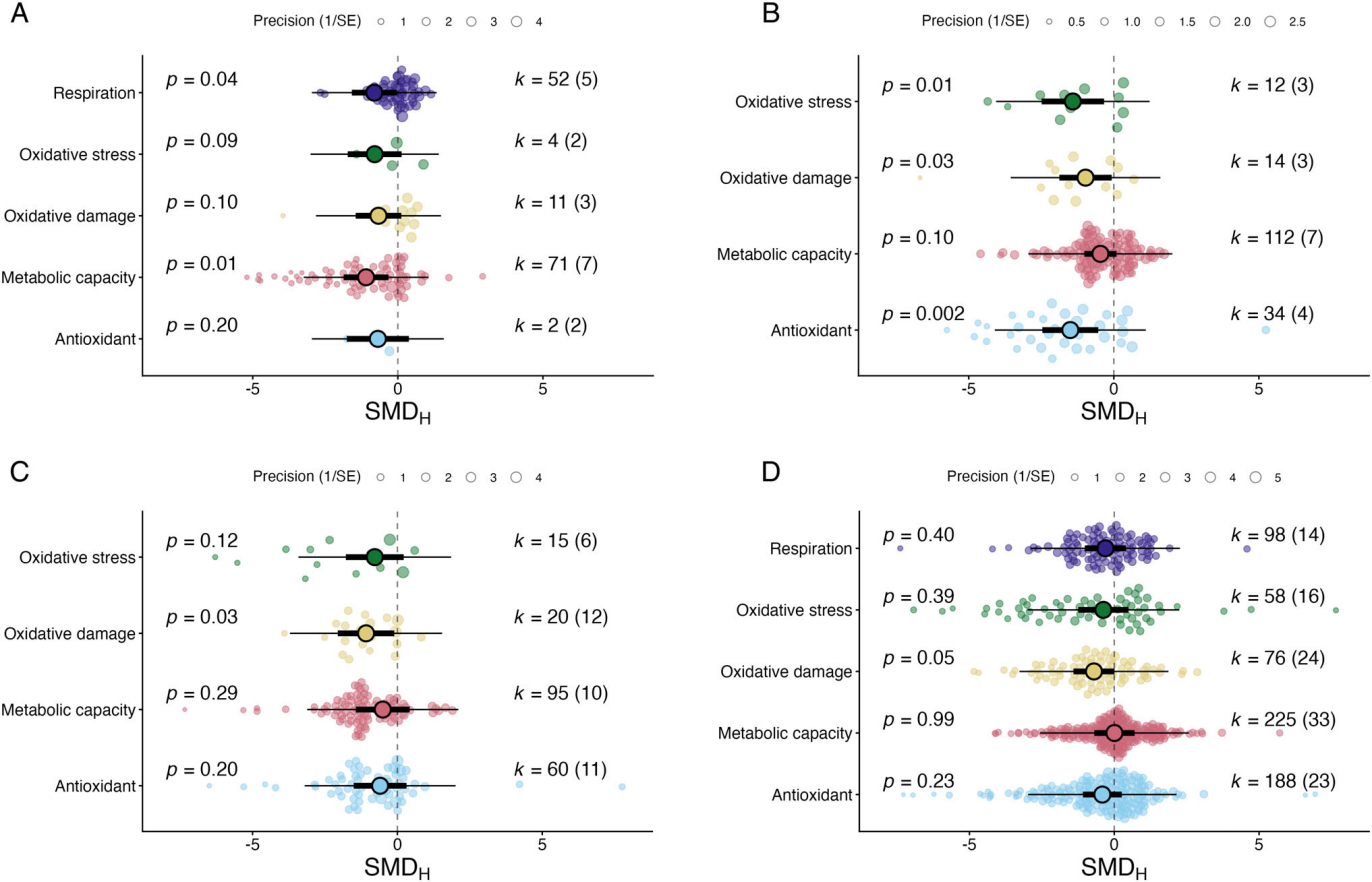
Overall meta‐analytic mean estimates across each functional trait category for each developmental stressor type (A) glucocorticoid exposure, (B) parental care deprivation, (C) psychological disturbance, and (D) nutritional imbalance. Mean estimates are shown as large points, with 95% confidence (thick black bars) and prediction (thin black bars). The number of effects (*k*) along with the number of studies (in parentheses) are shown provided for each trait category. The dashed lines represent no effect where the estimate of the standardized mean difference between treatment and control groups (SMD_H_) equals zero. Raw data (translucent bubbles) are weighted by their inverse sampling error (precision). Note that raw data is truncated for ease of visualisation.

The effects of glucocorticoid exposure during development on mitochondrial function appeared to be stronger in mammals than in birds and fish but there were no significant differences among taxonomic classes ($Q_{M_{2,137}}$ = 0.26, *P* = 0.77; Fig. 6A). Overall, mitochondrial respiratory function was suppressed in mammals (SMD_H_ = −1.15, 95% CI: −2.17 to −0.14, *P* = 0.03) and fish (SMD_H_ = −1.15, 95% CI: −2.55 to 0.26, *P* = 0.11), with weaker negative effects in birds (SMD_H_ = −0.49, 95% CI: −2.01 to 1.02, *P* = 0.52). Across classes, prenatal exposure to glucocorticoids had a larger negative impact on mitochondrial respiratory function than postnatal exposure (SMD_H_ = −1.09, 95% CI = −2.43 to 0.26); however, this effect was not significant (*P* = 0.11; Fig. 7A).

**Fig. 6:**
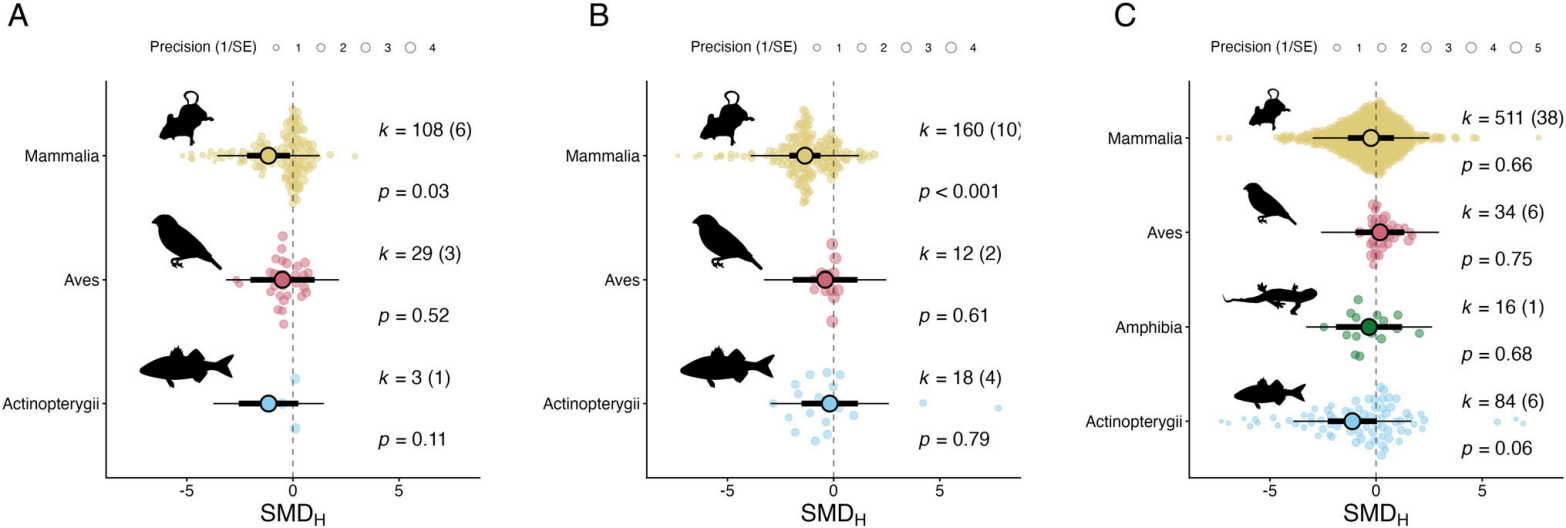
Overall meta‐analytic mean estimates for each taxonomic group (class) across developmental stressors (A) glucocorticoid exposure, (B) psychological disturbance, and (C) nutritional imbalance. Mean estimates are shown as large points, with 95% confidence (thick black bars) and prediction (thin black bars). The number of effects (*k*) along with the number of studies (in parentheses) are shown provided for each class. The dashed lines represent no effect where the estimate of the standardized mean difference between treatment and control groups (SMD_H_) equals zero. Raw data (translucent bubbles) are weighted by their inverse sampling error (precision). Raw data is truncated for ease of visualisation. The animal silhouettes are from PhyloPic (www.phylopic.org), have not been changed, and were contributed by David Liao (Mammalia, 2013; CC BY‐SA 3.0, https://creativecommons.org/licenses/by‐sa/3.0/), Andy Wilson (Aves, 2023; CC0 1.0), Denis Lafage, (Actinopterygii, 2020; CC BY 3.0, https://creativecommons.org/licenses/by/3.0/), and Beth Reinke (Amphibia, 2021; CC0 1.0).

**Fig. 7:**
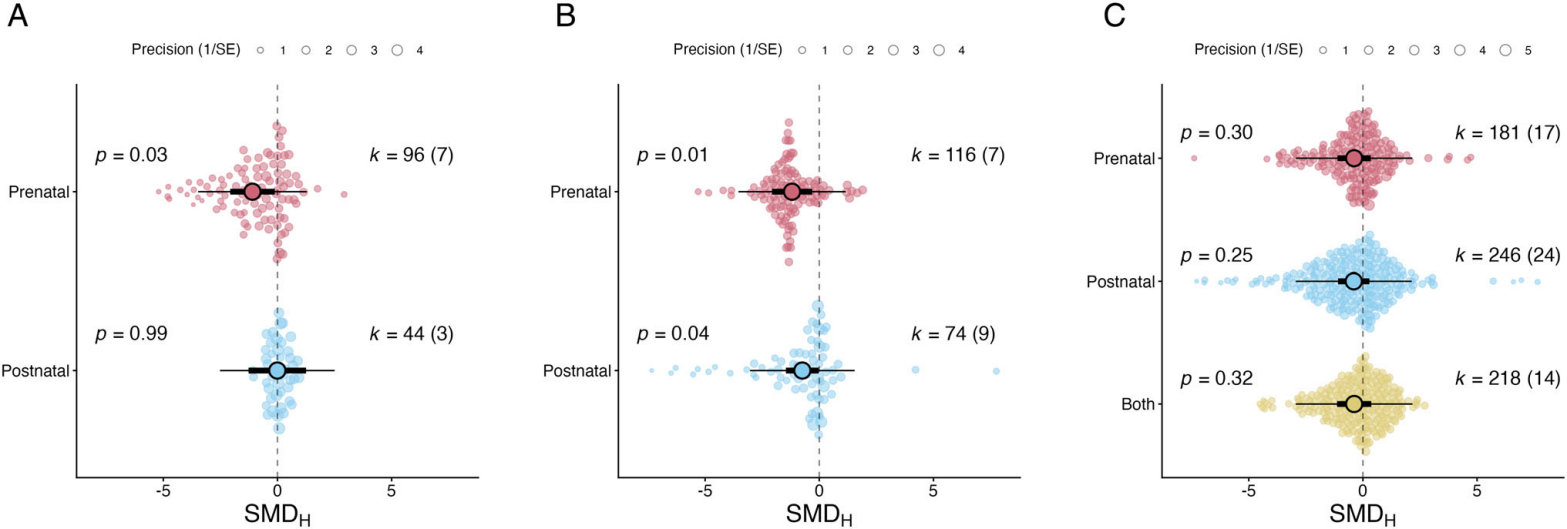
Overall meta‐analytic mean estimates different stages of manipulation (pre‐natal, post‐natal or both) across different developmental stressors (A) glucocorticoid exposure, (B) psychological disturbance, and (C) nutritional imbalance. Mean estimates are shown as large points, with 95% confidence (thick black bars) and prediction (thin black bars). The number of effects (*k*) along with the number of studies (in parentheses) are shown provided for each stage. The dashed lines represent no effect where the estimates of the standardized mean difference between treatment and control groups (SMD_H_) equals zero. Raw data (translucent bubbles) are weighted by their inverse sampling error (precision). Note that raw data is truncated for ease of visualisation.

### (2) Parental care deprivation – impacts on mitochondrial respiratory function

Parental care deprivation during development had a negative impact on mitochondrial respiratory function (SMD_H_ = −0.8, 95% CI = −1.4 to −0.21, *P* = 0.01; Fig. 4B). Effect heterogeneity was high (95% Prediction Intervals: −3.43 to 1.82; Table 3; see Tables S1 and S2), with 36.82% of the variation in effect size driven by differences among studies ($I_{\text{study}}^{2}$; Table 3). We found no evidence suggesting publication bias (See Supporting Information, Fig. S1, Appendix S3).

Parental care deprivation suppressed mitochondrial respiratory function across all major functional mitochondrial categories, but there were no significant differences between categories ($Q_{M_{3,168}}$ = 1.42, P = 0.24; Fig. 5B). However, parental care deprivation had larger negative effects on antioxidant capacity (SMD_H_ = −1.5, 95% CI: −2.46 to −0.54, P = 0.002), oxidative damage (SMD_H_ = −0.97, 95% CI: −1.88 to −0.07, P = 0.03) and oxidative stress (SMD_H_ = −1.41, 95% CI: −2.49 to −0.34, P = 0.01) compared to other categories. Metabolic capacity was also lower in treatment groups exposed to parental care deprivation compared to control groups. However, the effects were not significantly different from zero (SMD_H_ = −0.46, 95% CI: −1.02 to 0.09, P = 0.1; Fig. 5B). The studies included in our meta‐analysis that manipulated parental care were all conducted with mammals, and all studies necessarily applied treatments postnatally.

### (3) Psychological disturbance – impacts on mitochondrial respiratory function

Psychological disturbance during development had a negative impact on mitochondrial respiratory function (SMD_H_ = −0.8, 95% CI = −1.54 to −0.06, *P* = 0.03; Fig. 4C). However, there was evidence for publication bias, which if truly present, would change the overall meta‐analytic mean to SMD_H_ = 0.24 (95% CI = −0.6 to 1.08, *P* = 0.57; see Supporting Information, Fig. S1, Appendix S3). Effect heterogeneity was high (95% Prediction Intervals: −3.17 to 1.57; Table 3), with substantial variation across studies ($I_{\text{study}}^{2}$ = 57.25%; Table 3).

Exposure to psychological disturbance during development affected functional mitochondrial categories differently ($Q_{M_{3,186}}$ = 3.18, *P* = 0.04; Fig. 5C). Oxidative damage was most impacted by psychological disturbance (SMD_H_ = −1.08, 95% CI: −2.06 to −0.11, P = 0.03) and differed significantly from effects on antioxidants (SMD_H_ = −0.6, 95% CI: −1.51 to 0.31, P = 0.2) and metabolic capacity (SMD_H_ = −0.51, 95% CI: −1.44 to 0.42, *P* = 0.29; Fig. 5C).

In fish and birds, psychological disturbance was only manipulated postnatally. In contrast, psychological disturbance was also applied prenatally in studies with mammals. As such, there was some taxonomic bias. However, we did not find any significant differences among classes ($Q_{M_{2,187}}$ = 1.48, P = 0.23; Fig. 6B). Despite this, we found evidence that prenatal and postnatal disturbance stressors had different impacts on mitochondrial respiratory function. Overall, prenatal disturbance stressors had a larger negative impact on mitochondrial respiratory function (SMD_H_ = −1.19, 95% CI = −2.07 to −0.31, *P* = 0.01), whereas postnatal disturbance stressors had a smaller negative impact (SMD_H_ = −0.74, 95% CI = −1.46 to −0.02, *P* = 0.04; Fig. 7B), but these were not significantly different from each other (Contrast SMD_H_ = −0.45, 95% CI = −1.55 to 0.65, *P* = 0.42; Fig. 7B).

### (4) Nutritional imbalance – impacts on mitochondrial respiratory function

Nutritional imbalance during development had a weaker negative impact on mitochondrial respiratory function compared to other categories of developmental stressors (SMD_H_ = −0.38, 95% CI = −1.03 to 0.27, *P* = 0.25; Fig. 4D). We found evidence for publication bias (Fig. S1). Corrected meta‐analytic mean estimates, if publication bias were indeed present, may instead be slightly positive, but remain non‐significant (SMD_H_ = 0.17, 95% CI = −0.58 to 0.91, *P* = 0.66; see Supporting Information, Appendix S3). Effect heterogeneity was high (95% Prediction Intervals: −2.85 to 2.08; Table 3; see Tables S1 and S2), with variation being driven by differences between studies ($I_{\text{study}}^{2}$ = 8.57%), phylogeny ($I_{\text{phylogeny}}^{2}$ = 13.21%), and biological matrix ($I_{\text{matrix}}^{2}$ = 10.29%; Table 3).

Importantly, there was a taxonomic bias in the types of nutritional manipulations done. Studies that manipulated carbohydrates during development were only conducted with fish (Fig. 6C). The only nutritional manipulation that was conducted across all classes was total food availability. Whether animals received a deficit or surplus of food during development (i.e. under‐ or overnutrition) did not impact their mitochondrial respiratory function ($Q_{M_{1,643}}$ = 0.02, P = 0.9; Fig. 8A). There was weak evidence for an interaction between mitochondrial trait category and under/overnutrition ($Q_{M_{7,637}}$ = 1.4, *P* = 0.2). However, there was little evidence that mitochondrial respiratory function differed across functional trait categories generally (Fig. 5D). The best supported model, suggested that protein and fat manipulations did have a larger negative impact on mitochondrial respiratory function compared to carbohydrate manipulations but this effect was confounded with taxonomic class where only mammals had protein manipulations (protein contrast SMD_H_ = −1.32, 95% CI: −2.54 to −0.1, *P* = 0.03; fat contrast SMD_H_ = −1.1, 95% CI: −2.31 to 0.1, *P* = 0.07; Fig. 8B). There was no evidence that mitochondrial respiratory function was impacted by the developmental stage when nutritional manipulations were applied (Fig. 7C).

**Fig. 8:**
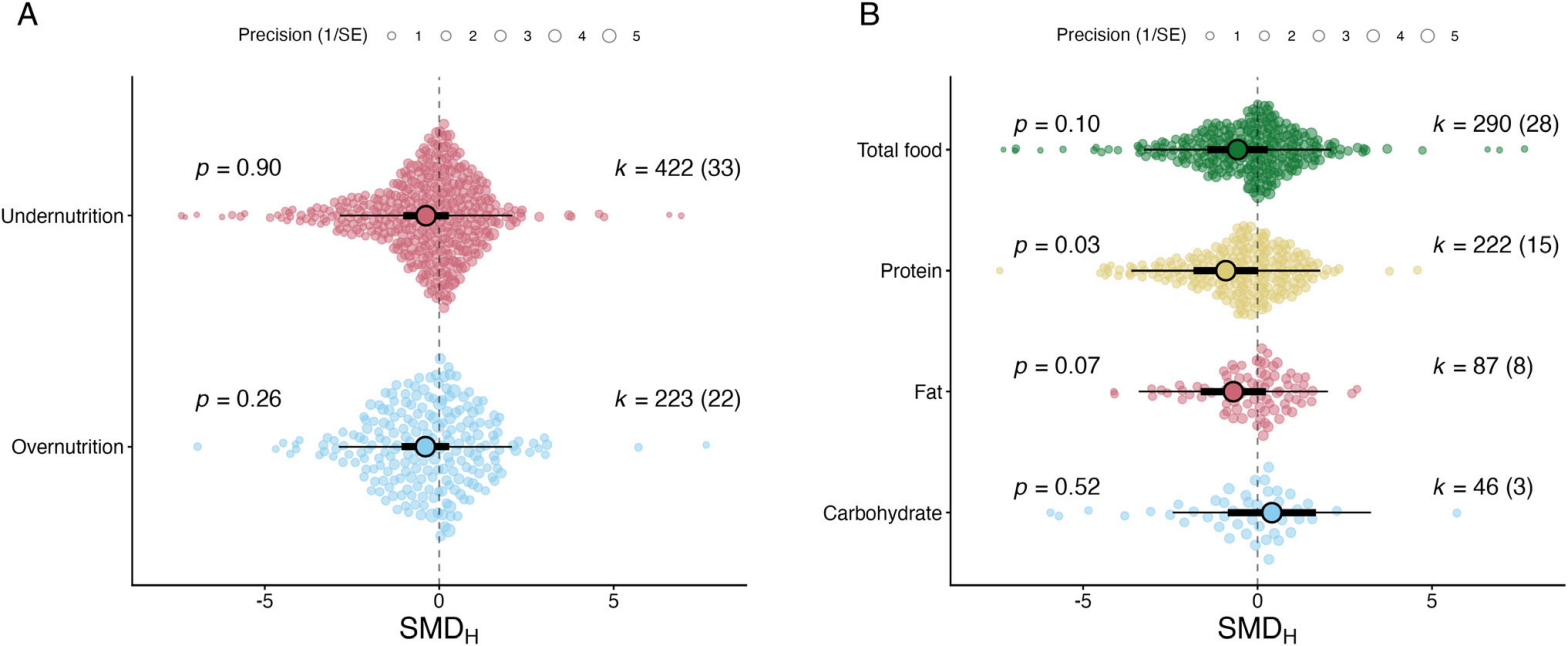
Overall meta‐analytic mean estimates for nutritional imbalance during (A) undernutrition (restricted) and overnutrition (excessive) and (B) for different nutrition types. Mean estimates are shown as large points, with 95% confidence (thick black bars) and prediction (thin black bars). The number of effects (*k*) along with the number of studies (in parentheses) are provided for each stressor type. The dashed lines represent no effect, where the estimates of the standardized mean difference between treatment and control groups (SMD_H_) equals zero. Raw data (translucent bubbles) are weighted by their inverse sampling error (precision). Note that raw data is truncated for ease of visualisation.

## IV. DISCUSSION

The developmental environment has long been recognized as a powerful force that shapes individual traits and evolutionary patterns (Uller, 2008; Monaghan, 2008; Mousseau & Fox, 1998). Mitochondrial respiratory function has been widely studied in medical research as a mechanism that links nutritional imbalances during early life to metabolic disorders and disease risk in later life (Grilo *et al*., 2021; Hoffman *et al*., 2021; Symonds *et al*., 2009). Additionally, ecologists and evolutionary biologists are increasingly incorporating metrics of mitochondrial respiratory function into studies aimed at understanding the mechanisms that link developmental stressors to phenotypic plasticity, individual fitness, and evolutionary patterns (Koch *et al*., 2021; Crino *et al*., 2024b; Hill, 2014; Dowling & Simmons, 2009; Monaghan, Metcalfe & Torres, 2009). However, our understanding of the general effects of developmental stressors on mitochondrial respiratory function across contexts has been hindered by the lack of quantitative synthesis. Here, we provide the first quantitative synthesis that tests the effects of developmental stressors on mitochondrial respiratory function across life‐stages and among vertebrate taxa. Based on patterns extracted from 86 published studies, we find that exposure to stressors during development generally decreases mitochondrial respiratory function, defined here as reduced oxygen consumption, lower antioxidant levels, and increased oxidative stress and oxidative damage. Although all categories of developmental stressors generally had negative effects on mitochondrial respiratory function, the magnitude of effects varied between categories of developmental stressors with some having larger effects than others (e.g. parental care deprivation compared to nutritional imbalance). Additionally, some mitochondrial traits were more strongly affected than others, although this varied between categories of developmental stressors. Our results can guide future research aimed at understanding how developmentally induced changes in mitochondrial respiratory function enable animals to acclimate to variable environmental conditions and affect individual fitness, life history strategies, and evolutionary patterns. Additionally, our results can inform future human health research aimed at developing predictive frameworks for understanding the impact of nutritional imbalances on the propensity for metabolic disorders and mitochondrial‐linked diseases.

### (1) Exposure to glucocorticoids, parental care deprivation, and psychological disturbances during development reduce mitochondrial respiratory function

We found that all categories of developmental stressors generally decreased mitochondrial respiratory function, although the degree of effects varied across categories of stressors. Exposure to glucocorticoids, parental care deprivation, and psychological disturbance during development had the strongest negative effects on mitochondrial respiratory function. Both parental care deprivation and psychological disturbances have been linked to acute HPA/I‐axis responses and long‐term changes in glucocorticoid physiology (e.g. Banerjee & Adkins‐Regan, 2011; Schmidt *et al*., 2004; Liu *et al*., 1997; Crino *et al*., 2013; Jafari *et al*., 2017). Although parental care deprivation and psychological disturbances could affect mitochondrial respiratory function through multiple physiological changes, it is likely that some of the effects are modulated through changes in glucocorticoid levels from acute responses to developmental treatments and/or programmatic changes to the HPA/I‐axis.

Glucocorticoids are often described as ‘stress’ hormones because of their role in regulating physiological and behavioural responses to disturbances, perturbations, and energetically demanding conditions (Sapolsky, Romero & Munck, 2000; Picard *et al*., 2018). Glucocorticoids and mitochondria are intrinsically linked because glucocorticoids are synthesized by mitochondria and modulate the availability of energy organisms need to cope with stressors through genomic and non‐genomic effects on mitochondrial respiratory function (Picard *et al*., 2018; Picard, Juster & McEwen, 2014; Lee *et al*., 2013; Midzak & Papadopoulos, 2016). Glucocorticoids bind to glucocorticoid receptors in the cytoplasm, forming complexes that are translocated into the nucleus where they alter expression of target nuclear genes associated with mitochondrial biogenesis and respiration (Lee *et al*., 2013; Lapp, Bartlett & Hunter, 2019; Kokkinopoulou & Moutsatsou, 2021). Additionally, mitochondria in some tissues contain glucocorticoid receptors (e.g. liver, brain; Du *et al*., 2009; Psarra & Sekeris, 2009). In these tissues, GC‐GR receptor complexes also translocate into mitochondria where they regulate mitochondrial gene expression through mitochondrial glucocorticoid responses elements (mGRE; Kokkinopoulou & Moutsatsou, 2021; Lee *et al*., 2013). Activation of mGREs can initiate or suppress mitochondrial genes encoding OXPHOS proteins and may result in faster mitochondrial responses to glucocorticoids compared to responses modulated by changes in nuclear gene expression (Haller, Mikics & Makara, 2008; Lee *et al*., 2013). Glucocorticoids also affect mitochondrial respiratory function through non‐genomic mechanisms such as cytosolic signalling pathways that occur relatively quickly (within minutes) compared to genomic modifications which can take up to several hours (Haller *et al*., 2008). Non‐genomic mechanisms of glucocorticoid action can affect mitochondrial respiratory function by inhibiting electron transfer between complexes in the electron transport system (ETS) and through changes in the structure of the mitochondrial membrane which results in increased proton leakage and reduced ATP production (Song & Buttgereit, 2006; Fujita *et al*., 2009). Overall, glucocorticoids regulate mitochondrial respiratory function through multiple genomic and non‐genomic pathways. Additionally, exposure to elevated levels of glucocorticoids during development, can change HPA/I‐axis function, ultimately resulting in sustained changes to endogenous glucocorticoids patterns which can, in turn, affect mitochondrial respiratory function (e.g. Nesan & Vijayan, 2016; Crino *et al*., 2022).

### (2) Nutritional imbalances during development and mitochondrial respiratory function

Nutritional imbalances can affect mitochondrial traits through rapid changes in mitochondrial dynamics (i.e. fission, fusion, and mitophagy), changes in hormone secretion (e.g. glucocorticoids, glucagon, norepinephrine), and transcriptional responses that occur over short and long‐term timescales (reviewed in Gao, Cantó & Houtkooper, 2014; Leverve, 2007; de Guia, Rose & Herzig, 2014). Functionally, these mechanisms allow organisms to adjust mitochondrial respiratory function to maintain metabolic homeostasis (Gao *et al*., 2014). However, our meta‐analysis showed weak (non‐significant) support for early life nutritional imbalances having negative effects on mitochondrial respiratory function. We found the effects of nutritional imbalances on mitochondrial respiratory function were influenced by the type of macronutrient manipulation with studies that manipulated protein (*P* = 0.03) and fat (*P* = 0.07) availability showing the largest negative effects on mitochondria respiratory function. However, this result was confounded by phylogeny because only studies with mammals manipulated protein availability. The effects of nutritional imbalance on mitochondrial respiratory function did not depend on whether manipulations restricted nutrients or supplied excessive nutrients. This result is consistent with past research demonstrating that both low and high birth weight (due to nutritional imbalances) predispose individuals to develop obesity and metabolic pathologies in later life (reviewed in Breton, 2013). This ‘U’‐shaped relationship between early life nutrition and adult metabolic pathologies underpinned the development of early hypotheses that proposed that perinatal nutrition permanently alters physiological systems resulting in adult metabolic pathologies (i.e. nutritional programming; De Boo & Harding, 2006; Fernandez‐Twinn & Ozanne, 2006; Gluckman & Hanson, 2004; Symonds *et al*., 2009). Several mechanisms have been linked to nutritional programming including epigenetic regulation of gene expression, changes to neuroendocrine pathways including the HPA‐axis and the hypothalamus‐adipose axis (a neuroendocrine pathway that regulates food intake and energy expenditure), and changes to mitochondrial respiratory function (Breton, 2013; Symonds *et al*., 2009). More so than in the other categories of developmental stressors, the distribution of effect sizes in studies that manipulated nutrition indicated that there was likely publication bias in the form of selective publication of studies with significant results. Notably, when we accounted for publication bias, the effect of nutritional imbalance on mitochondria function was positive, although the results were non‐significant. Together, our analysis highlights the importance of publishing non‐significant results as well as using animals from across taxa in experiments.

### (3) Timing of exposure to developmental stressors

Environmental perturbations and stressors are generally thought to induce stronger and more sustained organismal responses during earlier stages of development (Lindstrom, 1999; Monaghan, 2008; Skinner, 2014). Developing animals may be particularly sensitive to stressors experienced during the prenatal period that can exacerbate trade‐offs in energy allocation to physiological systems, organs, and structures (Monaghan, 2008). Such prenatal trade‐offs can result in sustained organismal effects both when postnatal compensatory growth or development is not possible and because of the costs associated with compensatory growth when it is possible (e.g. increased oxidative stress, reduced lifespan; Monaghan, 2008; Metcalfe & Monaghan, 2001). We found that exposure to glucocorticoids and psychological disturbances during prenatal development had larger negative impacts on mitochondrial respiratory function compared to exposure during the postnatal period. Prenatal stressors likely have strong effects on mitochondrial respiratory function due to epigenetic modifications that are particularly important for shaping early developmental pathways (Skinner, 2014; Kundakovic & Jaric, 2017). Epigenetic marks that occur during the prenatal period are mostly maintained throughout development and are less susceptible to environmental changes later in life (Skinner, 2014). However, postnatal and adult conditions can attenuate the long‐term effects of some prenatal stressors (e.g. Koo *et al*., 2003), highlighting the importance of longitudinal studies in understanding the long‐term organismal effects of developmental stressors. In contrast to the strong effects of prenatal glucocorticoids and psychological disturbances on mitochondrial respiratory function, we found no evidence that developmental stage (prenatal, postnatal, or combined prenatal and postnatal) impacted the effects of nutritional imbalances on mitochondrial respiratory function. However, nutritional imbalance had an overall weaker impact on mitochondrial respiratory function compared to exposure to glucocorticoids and psychological disturbance during development.

### (4) Potential effects of developmentally induced changes in mitochondrial respiratory function on organismal function and fitness

Our results suggest that developmental stressors generally reduce mitochondrial respiratory function by reducing mitochondrial respiration, antioxidant levels, and metabolic capacity and increasing oxidative stress and oxidative damage. However, reduced mitochondrial respiratory function (as defined here) does not necessarily result in maladaptive organismal responses. In some environments, reduced mitochondrial respiratory function could induce organismal changes that increase survival and reproduction. For example, proton conductance of the inner mitochondria membrane affects the rate that protons ‘leak’ into the mitochondrial matrix, resulting in reduced ATP and ROS production and increased heat production (i.e. increased uncoupling; Brand, 2000; Ricquier & Bouillaud, 2000). Greater mitochondrial uncoupling has been linked to longer lifespans and may be an important cellular mechanism underlying variable life‐history strategies (i.e. the “uncoupling to survive” hypothesis; Speakman *et al*., 2004; Yu & Chung, 2006; Brand, 2000). In our meta‐analysis, greater mitochondrial uncoupling was classed as reduced mitochondrial respiratory function because greater uncoupling reduces ATP production. However, in some contexts, increased uncoupling could increase evolutionary fitness through adaptive thermogenic responses (e.g. *via* changes in brown adipose tissue), variable investment in life history traits, and increased lifespan (Mookerjee *et al*., 2010; Mozo *et al*., 2005; Koch *et al*., 2021; Criscuolo *et al*., 2005). Future studies that test the effects of mitochondrial function on organismal function over lifespan and in biologically relevant contexts (e.g. matched *versus* unmatched prenatal/postnatal environments) will help uncover the adaptive significance of developmentally induced changes in mitochondrial respiratory function.

### (5) Limitations and future directions

One of our original aims was to evaluate how sex influences the effects of developmental stressors on mitochondrial respiratory function. However, we were unable to test for sex differences given the relatively small number of studies that included females as test subjects. In the studies included in our meta‐analysis, only 29.3% of effects included females and only 14.6% of total effects were presented for females (as opposed to grouping results for both sexes). In general, studies may focus preferentially on males because mitochondrial respiratory function can be affected by sex steroids (Velarde, 2014; Yin *et al*., 2021) and females (especially mammalian females) typically experience more fluctuations in sex steroids compared to males. Additionally, studies may focus on male test subjects when the motivation of the study arises from an interest in a pathology that afflicts males more than females (e.g. Hsu *et al*., 2018; Wang *et al*., 2023; Taylor & Turnbull, 2005). However, recent reviews of studies in rats and humans identified sex‐specific differences in several aspects of mitochondrial respiratory function including ROS production, respiratory capacity, and mitochondrial content (Ventura‐Clapier *et al*., 2017; Junker *et al*., 2022b). Testing sex‐specific responses in mitochondrial research will expand our ability to make inferences about how mitochondrial physiology can drive large scale ecological and evolutionary patterns. Even in experiments that do not aim to test sex effects, accounting for sex differences can increase the accuracy of research results by allowing researchers to account for sex‐specific variation in mitochondrial respiratory function (Junker, Juster & Picard, 2022a). Finally, accounting for sex in studies with humans can enhance our understanding of how mitochondrial respiratory function influences human health and the propensity for metabolic pathologies (Junker *et al*., 2022a; Ventura‐Clapier *et al*., 2017).

We included studies from 21 species across 13 orders. However, most studies (74.4%) were conducted in four species of mammals (*Mus musculus*, *Rattus norvegicus*, *Ovis aries* and *Capra hircus*). One category of developmental stressors included in our meta‐analysis (parental care deprivation) is implicitly taxonomically biased because, although widespread, parental care is not present in all taxonomic groups (Kokko & Jennions, 2012) and taxonomic bias in parental care research has been demonstrated elsewhere (Stahlschmidt, 2011). We also detected evidence of taxonomic bias in two other categories of developmental stressors in our meta‐analysis (nutritional imbalance and psychological disturbance). Studies that manipulate nutrient availability may also experience implicit taxonomic biases because some macronutrient manipulations may be more relevant for some taxonomic groups than others or it may be logistically impractical to manipulate specific macronutrient availability in certain diets (e.g. carbohydrates in a granivorous bird). Although there are many benefits for research that focuses on model species, it is unclear if findings from studies with mammals (and specifically laboratory rodents) can be broadly extrapolated across other taxa. Extending studies to include a greater diversity of taxonomic groups with different life history strategies will enable us to better understand how developmental conditions affect mitochondrial respiratory function.

## V. CONCLUSIONS

(1) There is a growing appreciation for the role of mitochondria in shaping individual traits which scale up to affect population dynamics and evolutionary and ecological patterns. Understanding how conditions like developmental stressors shape mitochondrial respiratory function across life is critical for understanding how constraints and trade‐offs influence physiological responses that allow organisms to cope with environmental conditions and drive population‐level responses.
(2) The effects of developmental stressors on individual traits have been widely documented across taxa. However, our understanding of how developmental stressors shape individual fitness and evolutionary outcomes has been hindered by a lack of understanding of the cellular mechanisms that link developmental stressors to variable trait expression.
(3) Here, we show that three major categories of developmental stressors (glucocorticoid exposure, psychological disturbance, and parental care deprivation) generally decrease mitochondrial respiratory function across taxa.
(4) Our results suggest that changes in mitochondrial respiratory function may link developmental stressors to changes in organismal traits and individual fitness and help shed light on the constraints that influence evolutionary outcomes.
(5) Finally, understanding the role of the developmental environment in shaping mitochondrial respiratory function across life is critical for understanding the role of mitochondrial dysfunction in human disease and pathology.

## Supporting information


**Appendix S1.** Key word search terms.
**Appendix S2.** Papers included in meta‐analysis.
**Appendix S3.** Exploratory statistics – publication bias and extreme effects.
**Table S1.** Exploratory statistics – heterogeneity analyses (mean‐scaled heterogeneity metric).
**Table S2.** Exploratory statistics – heterogeneity analyses (robust heterogeneity metric).
**Fig. S1.** Publication bias funnel plots.

**Appendix S4.** Data file.

## Data Availability

Data associated with this paper can be found in the Supporting Information (Appendix S4) and at: https://doi.org/10.5281/zenodo.17290931 and https://github.com/daniel1noble/mito_meta_stress.
