# Supplementary material for: How developmental stressors affect mitochondrial respiratory function: a systematic review and meta‐analysis
Source: Biol Rev Camb Philos Soc. 2026 Jul 14;101(6):2756–79. doi: 10.1002/brv.70199 (PMC13736821; doi:10.1002/brv.70199)
Supplement: Supplementary file 1 [file BRV-101-2756-s002.docx]

**Supporting information: How developmental stressors affect mitochondrial respiratory function: a systematic review and meta-analysis**

Ondi L. Crino, Christopher R. Friesen, Geoffrey E. Hill, Dalton C. Leibold, Pablo Recio, Karine Salin, Kristoffer H. Wild, Daniel W.A. Noble

**Appendix S1. Key word search terms.**

We searched titles, abstracts, and author keywords for the following search string: ((development* OR prenatal OR postnatal OR nestling* OR pup* OR egg* OR embryo* OR juvenile* OR hatchling* OR nestling* OR fetal OR fledgling* OR fry OR larva* OR ova OR ovum OR “early life”) AND (glucocorticoid* OR corticosterone OR cortisol OR stress* OR parental care OR maternal care OR paternal care OR weather OR temperature* OR nutrition* OR brood size OR litter size OR clutch size OR anthropogenic*) AND (“mitochondria* function” OR “mitochondria* biogenesis” OR RCR OR “ATP/O ratio” OR P/O ratio OR ROS OR ROMS OR LPO OR “oxygen consumption” OR “mitochondria* density” OR “mitochondria* number” OR “mitochondria* DNA damage” OR “mtDNA damage” OR “oxidative damage” OR “cytochrome c oxidase” OR CcO OR “uncoupling protein” OR UCP1) NOT (plant* OR leaf* OR leave* OR human* OR smoking OR men OR women OR child*))

**Appendix S2. Papers included in meta-analysis.**

Abelaira, H. M., Veron, D. C., De Moura, A. B., Carlessi, A. S., Borba, L. A., Botelho, M. E. M., Andrade, N. M., Martinello, N. S., Zabot, G. C., Joaquim, L., Biehl, E., Bonfante, S., Budni, J., Petronilho, F., Quevedo, J. & Réus, G. Z. (2021). Sex differences on the behavior and oxidative stress after ketamine treatment in adult rats subjected to early life stress. *Brain Research Bulletin* **172**, 129–138.

Alfaradhi, M. Z., Fernandez-Twinn, D. S., Martin-Gronert, M. S., Musial, B., Fowden, A. & Ozanne, S. E. (2014). Oxidative stress and altered lipid homeostasis in the programming of offspring fatty liver by maternal obesity. *American Journal of Physiology-Regulatory Integrative and Comparative Physiology* **307**, R26–R34.

Almeida, D. L., Moreira, V. M., Cardoso, L. E., Ferreira, M. J. R., Pavanelo, A., Ribeiro, T. A., Franco, C. C. D., Tófolo, L. P., Peres, M. N. C., Ribeiro, M. V. G., Ferreira, A. R. O., Gomes, R. M., Miranda, R. A., Trevenzoli, I. H., Armitage, J. A., Palma-Rigo, K. & Mathias, P. C. D. (2022). Lean in one way, in obesity another: effects of moderate exercise in brown adipose tissue of early overfed male Wistar rats. *International Journal of Obesity* **46**, 137–143.

Alonso-Alvarez, C., Bertrand, S., Faivre, B. & Sorci, G. (2007). Increased susceptibility to oxidative damage as a cost of accelerated somatic growth in zebra finches. *Functional Ecology* **21**, 873–879.

Amini-Khoei, H., Mohammadi-Asl, A., Amiri, S., Hosseini, M. J., Momeny, M., Hassanipour, M., Rastegar, M., Haj-Mirzaian, A., Haj-Mirzaian, A., Sanjarimoghaddam, H., Mehr, S. E. & Dehpour, A. R. (2017). Oxytocin mitigated the depressive-like behaviors of maternal separation stress through modulating mitochondrial function and neuroinflammation. *Progress in Neuro-Psychopharmacology & Biological Psychiatry* **76**, 169–178.

Banet, A. I., Healy, S. J., Eliason, E. J., Roualdes, E. A., Patterson, D. A. & Hinch, S. G. (2019). Simulated maternal stress reduces offspring aerobic swimming performance in Pacific salmon. *Conservation Physiology* **7**, coz095.

Banqueri, M., Gutiérrez-Menéndez, A., Méndez, M., Conejo, N. M. & Arias, J. L. (2021). Early life stress due to repeated maternal separation alters the working memory acquisition brain functional network. *Stress-the International Journal on the Biology of Stress* **24**, 87–95.

Banqueri, M., Martínez, J. A., Prieto, M. J., Cid-Duarte, S., Méndez, M. & Arias, J. L. (2019). Photobiomodulation rescues cognitive flexibility in early stressed subjects. *Brain Research* **1720**, 146300.

Bernal, A. B., Vickers, M. H., Hampton, M. B., Poynton, R. A. & Sloboda, D. M. (2010). Maternal undernutrition significantly impacts ovarian follicle number and increases ovarian oxidative stress in adult rat offspring. *PLoS ONE* **5**, e15558.

Braz, G. R. F., da Silva, A.I., Silva, S. C. A., Pedroza, A. A. S., de Lemos, M. D. T. B., de Lima, F. A. S., Silva, T. L. A. & Lagranha, C. J. (2020a). Chronic serotonin reuptake inhibition uncouples brown fat mitochondria and induces beiging/browning process of white fat in overfed rats. *Life Sciences* **245**, 117307.

Braz, G. R. F., Silva, S. C. D., Pedroza, A. A. D., de Lemos, M. D., de Lima, F. A., da Silva, A. I. & Lagranha, C. J. (2020b). Fluoxetine administration in juvenile overfed rats improves hypothalamic mitochondrial respiration and REDOX status and induces mitochondrial biogenesis transcriptional expression. *European Journal of Pharmacology* **881**, 173200.

Budge, H., Dandrea, J., Mostyn, A., Evens, Y., Watkins, R., Sullivan, C., Ingleton, P., Stephenson, T. & Symonds, M. E. (2003). Differential effects of fetal number and maternal nutrition in late gestation on prolactin receptor abundance and adipose tissue development in the neonatal lamb. *Pediatric Research* **53**, 302–308.

Burness, G. P., Mcclelland, G. B., Wardrop, S. L. & Hochachka, P. W. (2000). Effect of brood size manipulation on offspring physiology: an experiment with passerine birds. *Journal of Experimental Biology* **203**, 3513–3520.

Cao, K., Lv, W. Q., Hu, S. Q., Gao, J., Liu, J. K. & Feng, Z. H. (2020). Punicalagin activates AMPK/PGC-1α/Nrf2 cascade in mice: the potential protective effect against prenatal stress. *Molecular Nutrition & Food Research* **64**, 2000312.

Cao, K., Zheng, A., Xu, J., Li, H., Liu, J., Peng, Y. H., Long, J. G., Zou, X., Li, Y., Chen, C., Liu, J. K. & Peng, Z. H. (2014). AMPK activation prevents prenatal stress-induced cognitive impairment: modulation of mitochondrial content and oxidative stress. *Free Radical Biology and Medicine* **75**, 156–166.

Casagrande, S., Stier, A., Monaghan, P., Loveland, J. L., Boner, W., Lupi, S., Trevisi, R. & Hau, M. (2020). Increased glucocorticoid concentrations in early life cause mitochondrial inefficiency and short telomere. *Journal of Experimental Biology* **223**, jeb222513.

Castro, C., Corraze, G., Pérez-Jiménez, A., Larroquet, L., Cluzeaud, M., Panserat, S. & Oliva-Teles, A. (2015). Dietary carbohydrate and lipid source affect cholesterol metabolism of European sea bass (*Dicentrarchus labrax*) juveniles. *British Journal of Nutrition* **114**, 1143–1156.

Castro, C., Peréz-Jiménez, A., Coutinho, F., Corraze, G., Panserat, S., Peres, H., Teles, A. O. & Enes, P. (2018). Nutritional history does not modulate hepatic oxidative status of European sea bass (*Dicentrarchus labrax*) submitted to handling stress. *Fish Physiology and Biochemistry* **44**, 911–918.

Chen, W. X., Yang, H., Yan, Q. X., Zhou, X. L., Tan, Z. L. & Wang, Z. Y. (2022). Effects of maternal feed intake restriction on the blood parameters, fatty acid profile and lipogenetic genes expression of perirenal fat in offspring kids. *Animal Reproduction Science* **238**, 106955.

Chen, Y. T., Hu, Y., Yang, Q. Y., Liu, X. D., Son, J. S., De Avila, J. M., Zhu, M. J. & Du, M. (2021). Embryonic exposure to hyper glucocorticoids suppresses brown fat development and thermogenesis via REDD1. *Science Bulletin* **66**, 478–489.

Chen, Y. T., Hu, Y., Yang, Q. Y., Son, J. S., Liu, X. D., De Avila, J. M., Zhu, M. J. & Du, M. (2020). Excessive glucocorticoids during pregnancy impair fetal brown fat development and predispose offspring to metabolic dysfunctions. *Diabetes* **69**, 1662–1674.

Claycombe, K. J., Roemmich, J. N., Johnson, L., Vomhof-Dekrey, E. E. & Johnson, W. T. (2015). Skeletal muscle Sirt3 expression and mitochondrial respiration are regulated by a prenatal low-protein diet. *Journal of Nutritional Biochemistry* **26**, 184–189.

Cossin-Sevrin, N., Hsu, B. Y., Marciau, C., Viblanc, V. A., Ruuskanen, S. & Stier, A. (2022). Effect of prenatal glucocorticoids and thyroid hormones on developmental plasticity of mitochondrial aerobic metabolism, growth and survival: an experimental test in wild great tits. *Journal of Experimental Biology* **225**, jeb243414.

Costantini, D. (2010). Effects of diet quality on growth pattern, serum oxidative status, and corticosterone in pigeons (*Columba livia*). *Canadian Journal of Zoology-Revue Canadienne De Zoologie* **88**, 795–802.

Costantini, D., Angeletti, D., Strinati, C., Trisolino, P., Carlini, A., Nascetti, G. & Carere, C. (2018). Dietary antioxidants, food deprivation and growth affect differently oxidative status of blood and brain in juvenile European seabass (*Dicentrarchus labrax*). *Comparative Biochemistry and Physiology A-Molecular & Integrative Physiology* **216**, 1–7.

Crino, O. L., Falk, S., Katsis, A. C., Kraft, F. L. O. H. & Buchanan, K. L. (2022). Mitochondria as the powerhouses of sexual selection: testing mechanistic links between development, cellular respiration, and bird song. *Hormones and Behavior* **142**, 105184.

Davies, K. L., Camm, E. J., Smith, D. J., Miles, J., Forhead, A. J., Murray, A. J. & Fowden, A. L. (2023). Developmental programming of mitochondrial substrate metabolism in skeletal muscle of adult sheep by cortisol exposure before birth. *Journal of Developmental Origins of Health and Disease* **14**, 77–87.

De Faria, C. D. P., Martinez, C. B. D., Takahashi, L. S., De Mello, M. M. M., Martins, T. P. & Urbinati, E. C. (2021). Modulation of the innate immune *Fish Physiology and Biochemistry* **47**, 895–905.

de Moura Freitas, C., do Nascimento, L. C. P., Braz, G. R. F., Andrade-Silva, S. C., Lima-Junior, N. C., de Araju Silva, T., Fernandes, M. P., Ferreira, D. J. S., Lagranha, C. J. (2019). Mitochondrial impairment following neonatal overfeeding: a comparison between normal and ischemic-reperfused hearts. *Journal of Cellular Biochemistry* **120**, 7341–7352.

Do Prado, C. H., Narahari, T., Holland, F. H., Lee, H. N., Murthy, S. K. & Brenhouse, H. C. (2016). Effects of early adolescent environmental enrichment on cognitive dysfunction, prefrontal cortex development, and inflammatory cytokines after early life stress. *Developmental Psychobiology* **58**, 482–491.

Dong, Y. Z., Yu, M. H., Wu, Y. L., Xia, T., Wang, L., Song, K., Zhang, C. X., Lu, K. L. & Rahimnejad, S. (2022). Hydroxytyrosol promotes the mitochondrial function through activating mitophagy. *Antioxidants* **11**, 893.

Feng, Z. H., Zou, X., Jia, H. Q., Li, X. S., Zhu, Z. L., Liu, X. B., Bucheli, P., Ballevre, O., Hou, Y. F., Zhang, W. G., Wang, J. K., Chen, Y. & Liu, J. K. (2012). Maternal docosahexaenoic acid feeding protects against impairment of learning and memory and oxidative stress in prenatally stressed rats: possible role of neuronal mitochondria metabolism. *Antioxidants & Redox Signaling* **16**, 275–289.

Ferreira, D. J. S., Pedroza, A. A. D., Braz, G. R. F., da Silva, R. C., Lima, T. A., Fernandes, M. P., Doi, S. Q. & Lagranha, C. J. (2016a). Mitochondrial bioenergetics and oxidative status disruption in brainstem of weaned rats: immediate response to maternal protein restriction. *Brain Research* **1642**, 553–561.

Ferreira, D. S., Liu, Y. R., Fernandes, M. P. & Lagranha, C. J. (2016b). Perinatal low-protein diet alters brainstem antioxidant metabolism in adult offspring. *Nutritional Neuroscience* **19**, 369–375.

Gallagher, E. A. L., Newman, J. P., Green, L. R. & Hanson, M. A. (2005). The effect of low protein diet in pregnancy on the development of brain metabolism in rat offspring. *Journal of Physiology-London* **568**, 553–558.

Ghosh, S., Banerjee, K. K., Vaidya, V. A. & Kolthur-Seetharam, U. (2016). Early stress history alters serum insulin-like growth factor-1 and impairs muscle mitochondrial function in adult male rats. *Journal of Neuroendocrinology* **28**, 10.111/jne.12397.

Giordano, M., Costantini, D. & Tschirren, B. (2015). Sex-specific effects of prenatal and postnatal nutritional conditions on the oxidative status of great tit nestlings. *Oecologia* **177**, 123–131.

Gnanalingham, M. G., Mostyn, A., Symonds, M. E. & Stephenson, T. (2005). Ontogeny and nutritional programming of adiposity in sheep: potential role of glucocorticoid action and uncoupling protein-2. *American Journal of Physiology-Regulatory, Integrative and Comparative Physiology* **289**, R1407–R1415.

González-Pardo, H., Arias, J. L., Gómez-Lázaro, E., Taboada, I. L. & Conejo, N. M. (2020). Sex-specific effects of early life stress on brain mitochondrial function, monoamine levels and neuroinflammation. *Brain Sciences* **10**, 447.

González-Pardo, H., Arias, J. L., Vallejo, G. & Conejo, N. M. (2019). Environmental enrichment effects after early stress on behavior and functional brain networks in adult rats. *PLoS ONE* **14**, e0226377.

Gutierrez-Menendez, A., Banqueri, M., Mendez, M. & Arias, J. L. (2020). How does maternal separation affect the cerebellum? Assessment of the oxidative metabolic activity and expression of the c-Fos protein in male and female rats. *Cerebellum* **19**, 68–77.

Hellgren, L. I., Jensen, R. I., Waterstradt, M. S. G., Quistorff, B. & Lauritzen, L. (2014). Acute and perinatal programming effects of a fat-rich diet on rat muscle mitochondrial function and hepatic lipid accumulation. *Acta Obstetricia Et Gynecologica Scandinavica* **93**, 1170–1180.

Hsu, C. N., Lin, Y. J., Lu, P. C. & Tain, Y. L. (2018). Maternal resveratrol therapy protects male rat offspring against programmed hypertension induced by TCDD and dexamethasone exposures: is it relevant to aryl hydrocarbon receptor? *International Journal of Molecular Sciences* **19**, 2459.

Jia, N., Sun, Q. R., Su, Q., Dang, S. K. & Chen, G. M. (2016). Taurine promotes cognitive function in prenatally stressed juvenile rats via activating the Akt-CREB-PGC1α pathway. *Redox Biology* **10**, 179–190.

Jorgensen, W., Gam, C., Andersen, J. L., Schjerling, P., Scheibye-Knudsen, M., Mortensen, O. H., Grunnet, N., Nielsen, M. O. & Quistorff, B. (2009). Changed mitochondrial function by pre- and/or postpartum diet alterations in sheep. *American Journal of Physiology-Endocrinology and Metabolism* **297**, E1349–E1357.

Jousse, C., Muranishi, Y., Parry, L., Montaurier, C., Even, P., Launay, J. M., Carraro, V., Maurin, A. C., Averous, J., Chaveroux, C., Bruhat, A., Mallet, J., Morio, B. & Fafournoux, P. (2014). Perinatal protein malnutrition affects mitochondrial function in adult and results in a resistance to high fat diet-induced obesity. *PLoS ONE* **9**, e104896.

Khamoui, A. V., Desai, M., Ross, M. G. & Rossiter, H. B. (2018). Sex-specific effects of maternal and postweaning high-fat diet on skeletal muscle mitochondrial respiration. *Journal of Developmental Origins of Health and Disease* **9**, 670–677.

Khoa, T. N. D., Waqalevu, V., Honda, A., Shiozaki, K. & Kotani, T. (2020). Comparative study on early digestive enzyme activity and expression in red sea bream (*Pagrus major*) fed on live feed and micro-diet. *Aquaculture* **519**, 734721.

Li, X., Sun, H. L., Zhu, Z. L. & Li, H. (2014). The reduction of nNOS and ROS associated with decreased Ca2+ in hippocampus of prenatally stressed female offspring. *Neurochemical Journal* **8**, 282–288.

Maurer, A. D. & Reimer, R. A. (2011). Maternal consumption of high-prebiotic fibre or -protein diets during pregnancy and lactation differentially influences satiety hormones and expression of genes involved in glucose and lipid metabolism in offspring in rats. *British Journal of Nutrition* **105**, 329–338.

Monternier, P. A., Marmillot, V., Rouanet, J. L. & Roussel, D. (2014). Mitochondrial phenotypic flexibility enhances energy savings during winter fast in king penguin chicks. *Journal of Experimental Biology* **217**, 2691–2697.

Nascimento, L., Freitas, C. M., Silva, R., Leite, A. C. R., Silva, A. B., Da Silva, A. I., Ferreira, D. S., Pedroza, A. A., Maia, M. B. S., Fernandes, M. P. & Lagranha, C. (2014). The effect of maternal low-protein diet on the heart of adult offspring: role of mitochondria and oxidative stress. *Applied Physiology Nutrition and Metabolism* **39**, 880–887.

Noguera, J. C., Monaghan, P. & Metcalfe, N. B. (2015). Interactive effects of early and later nutritional conditions on the adult antioxidant defence system in zebra finches. *Journal of Experimental Biology* **218**, 2211–2217.

Ojha, S., Robinson, L., Yazdani, M., Symonds, M. E. & Budge, H. (2013). Brown adipose tissue genes in pericardial adipose tissue of newborn sheep are downregulated by maternal nutrient restriction in late gestation. *Pediatric Research* **74**, 246–251.

Oke, S. L., Sohi, G. & Hardy, D. B. (2020). Perinatal protein restriction with postnatal catch-up growth leads to elevated p66Shc and mitochondrial dysfunction in the adult rat liver. *Reproduction* **159**, 27–39.

Paiva, A. A., Faiad, J. Z., Taki, M. S., Reis, S. R. D., De Souza, L. M. I., Dos Santos, M. P., Chaves, V. E., Kawashita, N. H., De Oliveira, H. C. F., Raposo, H. F., Carneiro, E. M., Latorraca, M. Q., Gomes-Da-Silva, M. H. G. & Martins, M. S. F. (2012). A soyabean diet does not modify the activity of brown adipose tissue but alters the rate of lipolysis in the retroperitoneal white adipose tissue of male rats recovering from early-life malnutrition. *British Journal of Nutrition* **108**, 1042–1051.

Partadiredja, G., Worrall, S., Simpson, R. & Bedi, K. S. (2008). Pre-weaning undernutrition alters the expression levels of reactive oxygen species enzymes but not their activity levels or lipid peroxidation in the rat brain. *Brain Research* **1222**, 69–78.

Pedroza, A. A. S., Bernardo, E. M., Pereira, A. R., Silva, S. C. A., Lima, T. A., Freitas, C. D., Da Silva, J. C., Gomes, D. A., Ferreira, D. S. & Lagranha, C. J. (2021). Moderate offspring exercise offsets the harmful effects of maternal protein deprivation on mitochondrial function and oxidative balance by modulating sirtuins. *Nutrition Metabolism and Cardiovascular Diseases* **31**, 1622–1634.

Pijacka, W., Clifford, B., Tilburgs, C., Joles, J. A., Langley-Evans, S. & Mcmullen, S. (2015). Protective role of female gender in programmed accelerated renal aging in the rat. *Physiological Reports* **3**, e12342.

Prior, L. J., Velkoska, E., Watts, R., Cameron-Smith, D. & Morris, M. J. (2008). Undernutrition during suckling in rats elevates plasma adiponectin and its receptor in skeletal muscle regardless of diet composition: a protective effect? *International Journal of Obesity* **32**, 1585–1594.

Prokic, M. D., Petrovic, T. G., Despotovic, S. G., Vucic, T., Gavric, J. P., Radovanovic, T. B. & Gavrilovic, B. R. (2021). The effect of short-term fasting on the oxidative status of larvae of crested newt species and their hybrids. *Comparative Biochemistry and Physiology a-Molecular & Integrative Physiology* **251**, 110819.

Raap, T., Casasole, G., Pinxten, R. & Eens, M. (2016). Early life exposure to artificial light at night affects the physiological condition: an experimental study on the ecophysiology of free-living nestling songbirds. *Environmental Pollution* **218**, 909–914.

Reichert, S., Criscuolo, F., Zahn, S., Arrivé, M., Bize, P. & Massemin, S. (2015). Immediate and delayed effects of growth conditions on ageing parameters in nestling zebra finches. *Journal of Experimental Biology* **218**, 491–499.

Réus, G. Z., Fernandes, G. C., De Moura, A. B., Silva, R. H., Darabas, A. C., De Souza, T. G., Abelaira, H. M., Carneiro, C., Wendhausen, D., Michels, M., Pescador, B., Dal-Pizzol, F., Macêdo, D. S. & Quevedo, J. (2017). Early life experience contributes to the developmental programming of depressive-like behaviour, neuroinflammation and oxidative stress. *Journal of Psychiatric Research* **95**, 196–207.

Santana, D. F., Ferreira, D. S., Braz, G. R. F., Sousa, S. M. S., Silva, T. L. D., Gomes, D. A., Fernandes, M. P., Andrade-Da-Costa, B. L. & Lagranha, C. J. (2019). Maternal protein restriction in two successive generations impairs mitochondrial electron coupling in the progeny's brainstem of Wistar rats from both sexes. *Frontiers in Neuroscience* **13**, 203.

Sant'Anna, G. M. & Mortola, J. P. (2002). Thermal and respiratory control in young rats with altered caloric intake during postnatal development. *Respiratory Physiology & Neurobiology* **133**, 215–227.

Simoes-Alves, A. C., Costa-Silva, J. H., Barros, I. B., Da Silva, R. C., Vasconcelos, D. A. A., Vidal, H., Morio, B. & Fernandes, M. P. (2019). Saturated fatty acid-enriched diet-impaired mitochondrial bioenergetics in liver from undernourished rats during critical periods of development. *Cells* **8**, 335.

Sonei, N., Amiri, S., Jafarian, I., Anoush, M., Rahimi-Balaei, M., Bergen, H., Haj-Mirzaian, A. & Hosseini, M. J. (2017). Mitochondrial dysfunction bridges negative affective disorders and cardiomyopathy in socially isolated rats: pros and cons of fluoxetine. *World Journal of Biological Psychiatry* **18**, 39–53.

Stachowicz, A., Glombik, K., Olszanecki, R., Basta-Kaim, A., Suski, M., Lason, W. & Korbut, R. (2016). The impact of mitochondrial aldehyde dehydrogenase (ALDH2) activation by Alda-1 on the behavioral and biochemical disturbances in animal model of depression. *Brain Behavior and Immunity* **51**, 144–153.

Stone, V., August, P. M., Stocher, D. P., Klein, C. P., Couto, P. R. G., Silva, Y. D., Sagini, J. P., Salomon, T. B., Benfato, M. S. & Matté, C. (2016). Food restriction during pregnancy alters brain's antioxidant network in dams and their offspring. *Free Radical Research* **50**, 530–541.

Theys, N., Ahn, M. T., Bouckenooghe, T., Reusens, B. & Remacle, C. (2011). Maternal malnutrition programs pancreatic islet mitochondrial dysfunction in the adult offspring. *Journal of Nutritional Biochemistry* **22**, 985–994.

Theys, N., Bouckenooghe, T., Ahn, M. T., Remacle, C. & Reusens, B. (2009). Maternal low-protein diet alters pancreatic islet mitochondrial function in a sex-specific manner in the adult rat. *American Journal of Physiology-Regulatory Integrative and Comparative Physiology* **297**, R1516–R1525.

Toniazzo, A. P., Arcego, D. M., Lazzaretti, C., Mota, C., Schnorr, C. E., Pettenuzzo, L. F., Krolow, R., Moreira, J. C. F. & Dalmaz, C. (2019). Sex-dependent effect on mitochondrial and oxidative stress parameters in the hypothalamus induced by prepubertal stress and access to high fat diet. *Neurochemistry International* **124**, 114–122.

Velkoska, E., Cole, T. J. & Morris, M. J. (2005). Early dietary intervention: long-term effects on blood pressure, brain neuropeptide Y, and adiposity markers. *American Journal of Physiology-Endocrinology and Metabolism* **288**, E1236–E1243.

Welker, T. L. & Congleton, J. L. (2004). Oxidative stress in juvenile chinook salmon, *Oncorhynchus tshawytscha* (Walbaum). *Aquaculture Research* **35**(9), 881–887.

Wu, T. W., Huang, Y., Gong, Y. X., Xut, Y. J., Lu, J. Q., Sheng, H. & Ni, X. (2019). Treadmill exercise ameliorates depression-like behavior in the rats with prenatal dexamethasone exposure: the role of hippocampal mitochondria. *Frontiers in Neuroscience* **13**, 264.

Xiao, X. Q., Williams, S. M., Grayson, B. E., Glavas, M. M., Cowley, M. A., Smith, M. S. & Grove, K. L. (2007). Excess weight gain during the early postnatal period is associated with permanent reprogramming of brown adipose tissue adaptive thermogenesis. *Endocrinology* **148**, 4150–4159.

Yam, K. Y., Naninck, E. F. G., Abbink, M. R., La Fleur, S. E., Schipper, L., Van Den Beukel, J. C., Grefhorst, A., Oosting, A., Van Der Beek, E. M., Lucassen, P. J. & Korosi, A. (2017). Exposure to chronic early-life stress lastingly alters the adipose tissue, the leptin system and changes the vulnerability to western-style diet later in life in mice. *Psychoneuroendocrinology* **77**, 186–195.

Yang, F., Zhou, N., Zhu, X. L., Min, C. T., Zhou, W. & Li, X. N. (2021). n-3 PUFAs protect against adiposity and fatty liver by promoting browning in postnatally overfed *Journal of Nutritional Biochemistry* **93**, 108628.

Yang, H., Chen, N., Fan, L., Lin, X. J., Liu, J. C., You, Y. H., Zhong, Y., Chen, Y., Li, J. B. & Xiao, X. Q. (2022). Pre-weaning exposure to maternal high-fat diet is a critical developmental window for programming the metabolic system of offspring in mice. *Frontiers in Endocrinology* **13**, 816107.

Yap, K. N., Andreasen, V. A., Williams, A. S., Yamada, K. Y., Zikeli, S., Kavazis, A. N. & Hood, W. R. (2022). The persistent effects of corticosterone administration during lactation on the physiology of maternal and offspring mitochondria. *Journal of Experimental Biology* **225**, jeb244171.

Yu, N., Cao, X. H., Wang, Y. J., Kuang, S. W., Hu, J. B., Yang, Y., Xu, S. L., Zhang, M., Sun, Y. B., Gu, W. W. & Yan, X. J. (2020). Reduced stress responses byMS-222 in juvenile silver pomfret (*Pampus argeneus*). *Journal of the World Aquaculture Society* **51**, 1192–1207.

Zengin, H. (2021). The effects of feeding and starvation on antioxidant defence, fatty acid composition and lipid peroxidation in reared *Oncorhynchus mykiss* fry. *Scientific Reports* **11**, 16716.

Zhang, H., Mu, Z. B., Xu, L. M., Xu, G. F., Liu, M. & Shan, A. S. (2009). Dietary lipid level induced antioxidant response in manchurian trout, *Brachymystax lenok* (Pallas) larvae. *Lipids* **44**, 643–654.

Zheng, A., Li, H., Cao, K., Xu, J., Zou, X., Li, Y., Chen, C., Liv, J. K. & Feng, Z. H. (2015). Maternal hydroxytyrosol administration improves neurogenesis and cognitive function in prenatally stressed offspring. *Journal of Nutritional Biochemistry* **26**, 190–199.

Zhu, X. L., Du, S. S., Yan, Q. H., Min, C. T., Zhou, N., Zhou, W. & Li, X. N. (2021). Dietary curcumin supplementation promotes browning and energy expenditure in postnatal overfed rats. *Nutrition & Metabolism* **18**, 97.

**Appendix S3. Exploratory statistics – publication bias and extreme effects.**

While there was evidence for funnel asymmetry for the glucocorticoid (Fig. S1A), parental care deprivation (Fig. S1B), and psychological disturbance (Fig. S1C) datasets, this was not the case for the nutritional imbalance dataset (Fig. S1D). However, overall asymmetry is likely driven by small sample sizes in most of these datasets.

We more formally tested for publication bias by including the inverse effective sample size as a moderator in our multi-level meta-analysis (MLMA) models, which better controlled for sources of heterogeneity. Overall, we found no evidence for a significant slope, indicative of possible publication bias, for the glucocorticoid ($\beta$ = 1.06, 95% CI = –1.35 to 3.47, *P* = 0.39) or parental care deprivation ($\beta$ = –0.76, 95% CI = –3.92 to 2.41, *P* = 0.64) datasets. However, we did find evidence for a significant slope for the psychological disturbance model ($\beta$ = –3.94, 95% CI = –6.89 to –0.99, *P* = 0.01) and the nutritional imbalance model ($\beta$ = –2.09, 95% CI = –3.71 to –0.47, *P* = 0.01). Re-fitting models with the inverse effective sample size as a moderator suggested that, if publication bias was present, overall meta-analytic means would change to SMD_H_ = 0.24 (95% CI = –0.6 to 1.08, *P* = 0.57) for disturbance and SMD_H_ = 0.17 (95% CI = –0.58 to 0.91, *P* = 0.66) for nutrition.

In the nutritional imbalance and psychological disturbance datasets, we found two extreme effects that differed markedly from the majority of data (SMD_H_ > –20; Fig. S1). Careful data checking did not indicate any obvious data entry errors. We re-ran statistical models without the extreme effects to understand how they impacted the overall meta-analytic means. We found that removing the extreme effects had little impact on the overall meta-analytic means for nutritional imbalance (SMD_H_ = –0.38, 95% CI = –1.03 to 0.26, *P* = 0.25) and psychological disturbance (SMD_H_ = –0.78, 95% CI = –1.35 to –0.21, *P* = 0.01). As such, our overall conclusions remain unchanged.

**Table S1. Heterogeneity statistics for developmental stressor datasets based on the mean-scaled heterogeneity metric (CVH^2^; Yang et al. 2025), which can be interpreted as the proportion of heterogeneity relative to overall mean effect size.**

| $Stressor$ | $n_{effects}$ | $n_{species}$ | $n_{study}$ | $C{VH}_{total}^{2}$ | $C{VH}_{study}^{2}$ | $C{VH}_{phylogeny}^{2}$ | $C{VH}_{matrices}^{2}$ | $C{VH}_{obs}^{2}$ |
| --- | --- | --- | --- | --- | --- | --- | --- | --- |
| Glucocorticoids | 140 | 8 | 10 | 1.2 | 0.17 | 0.00 | 0.95 | 0.092 |
| Psychological disturbance | 190 | 8 | 16 | 2.0 | 1.46 | 0.28 | 0.00 | 0.301 |
| Nutritional imbalance | 645 | 15 | 51 | 9.9 | 1.06 | 1.64 | 1.28 | 5.949 |
| Parental care deprivation | 172 | 2 | 10 | 2.6 | 1.16 | – | – | 1.421 |

**Table S2. Heterogeneity statistics for developmental stressor categories based the robust heterogeneity metric (M^2^), which combines mean-scaled and variance-scaled metrics (see Yang *et al.*, 2025).**

| $Stressor$ | $n_{effects}$ | $n_{species}$ | $n_{study}$ | $M_{total}^{2}$ | $M_{study}^{2}$ | $M_{phylogeny}^{2}$ | $M_{matrix}^{2}$ | $M_{obs}^{2}$ |
| --- | --- | --- | --- | --- | --- | --- | --- | --- |
| Glucocorticoids | 140 | 8 | 10 | 0.55 | 0.078 | 0.00 | 0.43 | 0.042 |
| Psychological disturbance | 190 | 8 | 16 | 0.67 | 0.480 | 0.091 | 0.00 | 0.099 |
| Nutritional imbalance | 645 | 15 | 51 | 0.91 | 0.097 | 0.150 | 0.12 | 0.544 |
| Parental care deprivation | 172 | 2 | 10 | 0.72 | 0.324 | – | – | 0.397 |

**Fig.** **S1.** Funnel plots for each developmental stressor dataset (A) glucocorticoid exposure, (B) parental care deprivation, (C), psychological disturbance, and (D) nutritional imbalance. Each point represents an effect size (estimate of the standardized mean difference between treatment and control groups; SMD_H_) and its precision (1/SE). The dashed line represents no effect (i.e. SMD_H_ = 0).
